# Sustainable Biocontrol of Agave Vascular Wilt Using an Inactivated Mycelial Formulation from the Mangrove Endophyte *Talaromyces islandicus* M31

**DOI:** 10.1021/acsomega.6c01311

**Published:** 2026-04-21

**Authors:** Albert D. Patiño, Javier Plasencia, Sandip Das, Shabnam Hematian, Tania Raymundo, Carmina Montiel, Ricardo Valenzuela, Mario Figueroa

**Affiliations:** † Facultad de Química, 7180 Universidad Nacional Autónoma de México, Coyoacán, Ciudad de México 04510, Mexico; ‡ Department of Chemistry, Virginia Tech, Blacksburg, Virginia 24061, United States; § Escuela Nacional de Ciencias Biológicas, Instituto Politécnico Nacional, Miguel Hidalgo, Ciudad de México 11340, Mexico

## Abstract

The genus *Fusarium* comprises several plant pathogenic species, some of which are etiological agents of vascular wilt in agave plants, inducing systemic necrosis across the roots, caudex (cone), and foliar tissues. Such disease compromises host vigor and biomass productivity, ultimately reducing the organoleptic quality of fermented and distilled derivatives, including mezcal, tequila, and other distillates. Thus, as part of our ongoing bioprospecting efforts in unexplored areas of Mexico, the chemical study of the organic extract from a solid-state fermentation culture of the manglicolous endophyte fungus *Talaromyces islandicus* M31, isolated in the Punta Sur Ecological Park in Cozumel Island Biosphere Reserve, Mexico, led to the separation of eight anthraquinone derivatives active against the agave pathogens *Fusarium incarnatum* (CRT-153 and CRT-197), *Fusarium proliferatum* (CRT-142), and *Fusarium oxysporum* (CRT-098 and CRT-214). Among the molecules tested, the bisdihydroanthraquinone (–)-luteoskyrin (**6**) exhibited the strongest growth-inhibitory activity, which was also measured over a two-month span. Through macromolecule leakage assay and scanning electron microscopy, we found that (–)-luteoskyrin (**6**) disrupted membrane integrity in *F. incarnatum*. Furthermore, a simple formulation consisting of inactivated *T. islandicus* M31 mycelium was tested in vivo to control *Fusarium* infection in agave. The results suggest that (–)-luteoskyrin (**6**) acts synergistically with other components, resulting in remarkable anti-*Fusarium* activity and demonstrating its efficacy and advantages for pest management.

## Graphical abstract

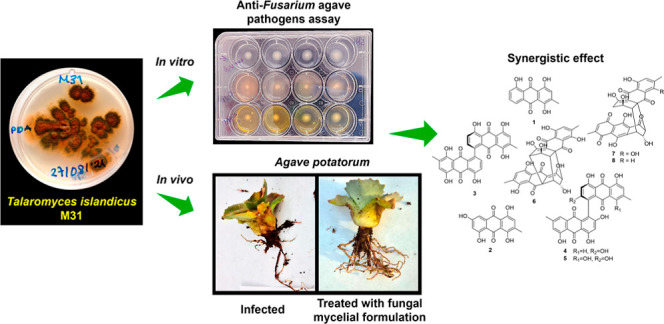

## Introduction

Fungal plant pathogens are one of the primary causes of crop diseases, resulting in significant yield losses and contributing to global food shortages.^1^ Among these, *Fusarium* species are one of the most ubiquitous economically important phytopathogens as they are the causal agents of several diseases in both monocot and dicot crops worldwide.^2^ In Mexico, crop losses due to *Fusarium* spp. infections are enormous, particularly in agave plantations. Most *Agave* spp. are native to Mexico, with 75% of the 200 known species found throughout the country. The economic importance of this plant is in the textile sector for fiber production and, most importantly, in the manufacture of fermented and distilled beverages such as tequila and mezcal.^3,4^ This pathogen affects 30–50% of all agave plantations, and its control mainly relies on the use of costly synthetic fungicides and soil disinfection by fumigants.^3,4^ However, the efficacy of these treatments is often lower than expected, plus the use of chemicals must be reduced because of adverse environmental effects and the generation of resistance strains, making the need for alternative control measures urgent.^2–4^

The search for biological control agents against *Fusarium* spp. has primarily focused on well-characterized genera such as *Trichoderma*, *Bacillus*, and *Pseudomonas*.^2^ These organisms mitigate wilt through mechanisms, including mycoparasitism, competition for nutrients, and the induction of systemic resistance in the host plant. However, despite these developments, many biocontrol strategies fail to translate from the laboratory to the field due to low stability or complex application methods, underscoring the need for robust, easily formulated agents.^2^ Another approach to address these problems is the discovery of control agents from natural sources, given their environmentally friendly properties (biodegradability and biocompatibility) and their potential for new modes of action. One of these strategies involves using endophytic microorganisms, which live within plant tissues without causing apparent damage or disease symptoms.^5^ In fact, they actively support their hosts in combating biotic (e.g., pathogen infection) and abiotic (e.g., drought, extreme temperatures, and salinity) stresses by producing a wide range of bioactive natural products and maintaining the harmony of different biogeochemical processes, sustaining nutritional status and ecological balance.^6^ These special characteristics make them an excellent model for studying bioactive compounds, which could be approached in medicine, biotechnology, and agriculture.^7,8^

Mangrove forests are among the most productive and diverse ecosystems worldwide, especially in tropical regions (e.g., Brazil, Indonesia, Australia, and Mexico), where they are most abundant.^9^ Mexico, which has the fourth-largest mangrove area in the world, covers more than 900,000 ha and hosts many endemic species, including microorganisms.^10^ However, bioprospecting on endophytic microorganisms from Mexican mangroves remains limited.^11^ Thus, in this study, a series of manglicolous endophytic fungi isolated from *Rhizophora mangle* collected in the Punta Sur Ecological Park in the Cozumel Island Biosphere Reserve, Mexico, were examined for their ability to produce metabolites that inhibit agave fungal pathogens. In particular, the fungus *Talaromyces islandicus* M31 exhibited the strongest activity against all of the *Fusarium* species. Although several isolates of this fungus have been studied for their production of amylases, phospholipases, dehydrogenases, and lyases,^12–15^ as plant growth promoters,^16,17^ for nanoparticle synthesis,^18–20^ as bioadsorbents of metals,^21^ and as producers of bioactive natural products,^22–30^ this species has never been explored for pest management. We hypothesize that the specific secondary metabolites of *T. islandicus* M31, particularly anthraquinone derivatives, can effectively inhibit agave pathogenic *Fusarium* species by disrupting their membranes. Thus, the objective of this study was to identify these bioactive compounds and evaluate an inactivated mycelial formulation as a sustainable in vivo treatment for agave vascular wilt.

## Experimental Section

### General Experimental Procedures

Optical rotation and circular dichroism data were measured using a PerkinElmer 343 polarimeter and a JASCO J-1500 spectrophotometer. NMR spectra were acquired on either a JEOL ECA-600 (600 MHz for ^1^H and 150 MHz for ^13^C) or a Varian INOVA 400 (400 MHz for ^1^H and 100 MHz for ^13^C) spectrometer in CDCl_3_, acetone-*d*_6_, or DMSO-*d*_6_. HRESIMS data were acquired using a Thermo Scientific Q Exactive Plus Hybrid Quadrupole-Orbitrap system equipped with an electrospray ionization (ESI) source and a HCD cell. Samples were introduced to the mass spectrometer via a UPLC system equipped with a BEH C_18_ column (1.7 μm, 50 × 2.1 mm i.d.). Separation by HPLC was performed on a Waters HPLC system equipped with a 2535 quaternary pump, a 2707 autosampler, and 2998 PDA and 2424 ELSD detectors, using Gemini C_18_ columns (5 μm, 110 Å, 250 × 4.6 mm i.d. for analytical and 5 μm, 110 Å, 250 × 21.2 mm i.d. for preparative). Flash chromatography was performed on a CombiFlash Rf+ Lumen system equipped with a PDA and ELSD detector and using RediSep Rf Gold 60 (40–60 μm) columns. Column chromatography (CC) was performed on a glass column packed with C_18_ silica gel (40–63 μm).

### Fungal Material

Endophytic fungi were isolated from *R. mangle* segments (i.e., branches, roots, and leaves) collected at the Punta Sur Ecological Park (20°17′34.4″ N, 86°57′47.4″ W) in the Cozumel Island Biosphere Reserve, Mexico, during May and October 2019.^31,32^ The climate in the study area is tropical with an average temperature of 25 °C and a mean annual precipitation of 1570 mm.^33^ The macromorphological and microscopic characteristics of the strains were examined using a Carl Zeiss model Stemi Dv4 stereomicroscope and a Nikon Eclipse 80i microscope. Molecular identification was performed following the methodology described by Raja et al.^34^ The sequences were assembled, submitted to NCBI BLAST for nucleotide homology searches against the GenBank database, and identified based on ≥99% sequence similarity (Supporting Information). The cultures are maintained at 4 °C at the fungal culture collection of the Department of Pharmacy at the Universidad Nacional Autónoma de México.

### Phytopathogens from Agave

Agave phytopathogens were donated by the Tequila Regulatory Council (CRT) of Mexico and identified as *Fusarium oxysporum* (CRT-098 and CRT-214), *Fusarium proliferatum* (CRT-142), and *Fusarium incarnatum* (CRT-153 and CRT-197) by PCR amplification and sequencing of the translation elongation factor 1α (TEF1α) and internal transcribed spacer (ITS) region and a search in the Fusarium ID database (https://www.fusarium.org/).^35^ All strains were isolated from *Agave tequilana* Weber var. *azul* plants showing wilt symptoms in various municipalities throughout the state of Jalisco, Mexico (Supporting Information).

### Fermentation, Extraction, and Isolation

Small pieces (1 × 1 cm^2^) of each axenic culture from the PDA plates were transferred to YESD medium (1% yeast extract, 2% soy peptone, 2% dextrose) and incubated for 5 days at 120 rpm. Subsequently, the liquid cultures were transferred to 250 mL Erlenmeyer flasks (2 flasks) containing rice medium (10 g of rice and 20 mL of deionized water) and maintained under a 12/12 h light–dark cycle for 21 days. After growth, the cultures were extracted with 60 mL of 1:1 MeOH–CHCl_3_, shaken at 100 rpm, and filtered. Then, 60 mL of CHCl_3_ and 120 mL of H_2_O were added to the filtrates and mixed. The organic layers were separated and dried under reduced pressure. The dry product was dissolved in 60 mL of 1:1 CH_3_CN–MeOH and defatted with the same volume of *n*-hexane. Defatted extracts were preserved at 4 °C until use.^36^

For *T. islandicus* M31, a scale-up culture (10×) was prepared, and its organic extract (10.2 g) was subjected to flash chromatography on a 55 g RediSep Rf Gold silica gel column (4 separations of 2.0 g each) using a gradient solvent system of *n*-hexane–CHCl_3_–AcOEt–CH_3_OH at a flow rate of 40 mL/min. A total of 22 fractions were collected and tested for antifungal activity. Among these, active fraction F5 (190.4 mg) was composed solely of pure compound **1**. Active fraction F6 (125.6 mg) was separated by preparative HPLC using a gradient from 40:60 to 100:0 of CH_3_CN-0.1% aqueous formic acid in 6 min at 21.24 mL/min (run time: 22 min), yielding additional amounts of compound **1** (6.8 mg, *t*_R_ = 9.4 min) and compound **3** (22.3 mg, *t*_R_ = 16.4 min). Active fraction F7 (40.2 mg) was fractionated via CC over C_18_ silica gel using a gradient from 75:25 to 100:0 of H_2_O–CH_3_CN, resulting in compound **2** (4.2 mg). Active fraction F15 (100.5 mg) was subjected to preparative HPLC using a gradient from 40:60 to 100:0 of CH_3_CN-0.1% aqueous formic acid in 10 min at 21.24 mL/min (run time: 22 min), yielding compound **4** (18.2 mg, *t*_R_ = 12.5 min), compound **5** (4.9 mg, *t*_R_ = 14.2 min), and compound **6** (26.3 mg, *t*_R_ = 9.5 min). Finally, active fraction F21 (85.6 mg) was subjected to preparative HPLC using a gradient from 60:40 to 100:0 of CH_3_CN-0.1% aqueous formic acid in 18 min at 4.72 mL/min (run time: 20 min), yielding compound **7** (15.6 mg, *t*_R_ = 9.8 min) and compound **8** (3.9 mg, *t*_R_ = 9.3 min).

### X-ray Crystallographic Analysis of **7**

Suitable single crystals of **7** for X-ray diffraction (Supporting Information) were obtained from a mixture of CH_3_CN–H_2_O (95:5). The crystallographic data and refinement parameters are provided in the Supporting Information. Briefly, all reflection intensities were measured at 100(2) K using a Bruker D8 Venture X-ray diffractometer equipped with a Photon III shutterless area detector with CuKα radiation (λ = 1.54178 Å). A preliminary set of unit cell parameters was harvested from 180 frames collected prior to full data collection. These parameters were then used for the full data collection at a detector distance of 4.2 cm. Data reduction, integration, and unit cell determination were performed using the APEX4 software package. The initial structure was solved using SHELXS, as implemented in APEX4. The structure was subsequently refined using Olex2 (v1.5) against *F* ^2^ by weighted full-matrix least-squares. All non-hydrogen atoms were refined anisotropically. Hydrogen atoms attached to carbon were placed in calculated positions using a riding model with the “HFIX” command. The hydrogen atoms bound to the oxygen were located in difference Fourier maps and refined with restrained geometry. Crystallographic data for compound **7** have been deposited with the Cambridge Crystallographic Data Centre (CCDC 2518651) and are available free of charge.

### Screening and Bioactivity-Guided Chemical Study

The fungal organic extracts and *T. islandicus* M31 fractions were tested in triplicate for their antiagave *Fusarium* pathogen activity using the poison-plate assay.^37^ Briefly, samples were dissolved in DMSO and added to reach a final concentration of 200 μg/mL to the PDA media before pouring the agar onto the plate. After solidification, a small plug (5 mm^2^) of the agave fungal pathogen was inoculated in the center of the plate and incubated for 5 days at 28 °C. Then, the pathogen radial growth was measured using Fiji software,^38^ and the % of inhibition was calculated using the following equation: $\%\;inhibition=\left(\frac{C-T}{C}\right)\times 100$ , where *C* and *T* refer to the radial mycelial diameter (in mm) of the fungal pathogen in the poisoned plates with DMSO (control) or with extract (treatment) (Supporting Information).

### Anti-*Fusarium* Activity of Pure Compounds

Pure compounds were tested for antifungal activity using the broth microdilution method, following the approved procedures published by the Clinical and Laboratory Standards Institute (CLSI) for antifungal susceptibility testing of filamentous fungi,^39^ with some modifications. The compounds were dissolved in DMSO to prepare stock solutions and were initially tested at final concentrations of 100 μg/mL and 10 μg/mL in PDB in triplicate. Each *Fusarium* spp. was grown on PDA for 5 to 7 days at 28 °C, and plates were flooded with sterile water to collect conidia. Conidia were counted in a hemocytometer to prepare cell suspensions adjusted to 1 × 10^5^ conidia/mL. Each concentration in 96-well plates was inoculated with 10^3^ conidia. Growth curves were generated by incubating at 28 °C for 72–96 h and monitoring OD_600nn_ with a microplate reader (Epoch) until reaching the stationary growth phase (Supporting Information). The compounds with the strongest activity at 10 μg/mL and 100 μg/mL were reevaluated in the same assay to determine their minimum inhibitory concentration (MIC), 50% inhibitory concentration (IC_50_), and fungistatic activity over a 2-month period. The latter assay was performed in 20 mL scintillation vials (1 mL assay volume) at the MIC and at 2× the MIC. Each week, an aliquot of 3 μL of the assayed medium was transferred to fresh PDA plates and incubated at 28 °C to observe fungal growth. In all assays, DMSO (1%) was used as a negative control, and ketoconazole (50 μg/mL) and/or nystatin (50 μg/mL) were selected as positive controls and model antifungal agents since their mechanisms of action are well-documented: nystatin is a natural product that induces membrane pore formation, and ketoconazole is a synthetic imidazole that inhibits ergosterol biosynthesis.

### Membrane Integrity Assay

The effect of luteoskyrin (**6**) on *F. incarnatum* (CRT-153 and CRT-197) cell membrane integrity was assessed by measuring the leakage of cellular macromolecules (nucleic acids and proteins) into the extracellular medium.^40^ Briefly, the pathogens were grown from conidia culture at 1 × 10^5^ conidia/mL in 2 mL of PDB for 48 h. Subsequently, the fungi were separated by centrifugation of the culture broth for 10 min at 6000 rpm, and the cell pellets were washed twice with PBS and resuspended in 1 mL of PBS. Then, the pellet was treated with **6** at its MIC and IC_50_ in triplicate, and after 24 h of incubation, the suspension was centrifuged at 10,000 rpm for 20 min to separate the cell-free supernatant. The released nucleic acids were quantified using a NanoDrop 2000 spectrophotometer (260 nm),^41^ and the protein was measured by the Bradford method (BSA curve; 595 nm).^42^ Nystatin and ketoconazole were used as positive controls.

### Fungal Mycelia Morphology

The effect of luteoskyrin (**6**) on *F. incarnatum* CRT-197 mycelial morphology was analyzed by a scanning electron microscope (SEM). For this purpose, sample preparation was performed according to standard protocols.^43^ Briefly, mycelia samples separated after treatment with **6**, in triplicate, were soaked in a buffer solution of 2.5% glutaraldehyde in 0.2 M cacodylate at pH 7.2 for 4–6 h at 4 °C. Then, samples were washed three times for 10 min each in 0.1 M sodium cacodylate buffer and postfixed in aqueous 1% osmium tetroxide (OsO_4_) for 2 h at 4 °C. The samples were washed again with the same buffer for 10 min and then dehydrated in an acetone gradient from 35% to 100%. Finally, the samples were transferred to a sample basket, placed in a critical-point dryer (CPD) for 90 min, and then mounted on stubs using double-sided tape and gold-coated for SEM imaging with a JEOL JSM-5410LV.

### Fungal Mycelial Formulation

A simple formulation comprising the inactivated *T. islandicus* M31 mycelium grown on rice medium (80 g of rice, 160 mL of deionized water, 18 days of culture in a 12/12 h light–dark cycle, and room temperature) was prepared by adding 80 mL of MeOH and ground in a mortar and pestle under liquid nitrogen, yielding 62.1 g of product. Complete inactivation of the formulation was confirmed by inoculating the product onto PDA plates and maintaining the plates under a 12/12 h light–dark cycle at 30 °C for 7 days (Supporting Information). The amount of (–)-luteoskyrin (**6**) in the formulation was determined by HPLC analysis (Supporting Information). Briefly, 500 mg of the fungal mycelial formulation was extracted by maceration with CHCl_3_–MeOH (1:1), using the same procedure as for the bioactivity-guided chemical study (see section Fermentation, Extraction, and Isolation), yielding 35.6 mg of crude extract. This extract was then analyzed by HPLC using an ELSD detector. Relative quantification was assessed by measuring the area under the curve (AUC) in triplicate of the peak at *t*_R_ 8.68 min, which matches the UV profile and retention time of pure compound **6**. Based on this analysis, the crude extract contains 52% of **6** (18.5 mg within 35.6 mg of extract), corresponding to a 3.7% concentration in the fungal mycelial formulation.

### In Vivo Anti-*Fusarium* Activity of Inactivated *T. islandicus* M31 Mycelium

The inactivated *T. islandicus* M31 mycelial formulation (fungal mycelial formulation) was tested in vivo to control infection by Fusarium pathogens in agave plants, following the protocol described by Nasir et al.^44^ with some modifications. Briefly, three-month-old *Agave potatorum* and *Agave angustifolia* plants were employed, and their roots were vigorously washed with sterile distilled water before being wounded with a needle and dipped into a spore solution (1 × 10^6^ conidia/mL) of *F. incarnatum* (CRT-153 and CRT-197) or with sterile distilled water. The plants were held at room temperature for 30 min before being repotted in a mixed substrate composed of tezontle (10%), vermiculite (80%), and peat moss (10%).^45^ Then, the *T. islandicus* M31 formulation was applied to the potted agave surface at 0.15 mg/cm^3^ or 0.75 mg/cm^3^ (25 mg and 125 mg in a plant pot of 16 cm^3^), maintaining the plants in the greenhouse (13–35 °C and humidity 15–64%), and 25 mL of water was added every week. Disease progression was monitored weekly for one month.^44,46^ All experiments were performed in triplicate, and sterile distilled water served as the negative control. After one month, pathogen colonization in agave roots was estimated by measuring growth on a selective medium for *Fusarium* (Nash and Snyder medium).^47^ For this purpose, several roots of each treated plant were disinfected, cut into fragments (5 mm^2^), and inoculated into agar plates. Then, the plates were incubated at 28 °C, and fungal growth was observed after 4 days. The percentage of fungal growth on the plate was determined by measuring the area using Fiji.^38^

### Molecular Networking of *T. islandicus* M31

The fungal extract was analyzed by ultrahigh-performance liquid chromatography-tandem high-resolution electrospray mass spectrometry (UPLC-HRESIMS/MS) using the previously described methodology.^48^ Raw data were preprocessed in MZmine 4.85 according to the following workflow: mass detection at MS1 and MS2, chromatogram builder, resolving, ^13^C isotope filtering, join aligner, feature finder, duplicate filter, feature height filter, MS2 filter, feature grouping, and ion identity networking.^49,50^ Features that had less than a 3-fold difference from the blank were removed. The group intensity threshold, the minimum highest intensity, and the minimum absolute height parameters were adjusted on the basis of the selected MS1 noise level. The resulting MS1 feature list was exported to Excel (.csv), and the MS2 feature data were exported as an.mgf file for feature analysis in the Global Natural Products Social v2 (GNPS2) platform. A molecular network was constructed using the feature-based molecular networking (FBMN) workflow in GNPS2 (https://gnps2.org/homepage),^51^ with edges filtered to have a cosine score above 0.65 and more than 5 matched peaks. The spectra in the network were compared to GNPS2 spectral libraries.^52^ For metabolites without automatic annotations, the chemical assignments were carried out using the CANOPUS tool from SIRIUS software, version 5.8.5, also using the NPClassifer system. Visualization and editing of the molecular networks were done using Cytoscape 3.10.4.^53^ Finally, the annotation of compounds was at confidence levels 1 and 2 according to the Metabolomics Standards Initiative and exact mass accuracy of <5 ppm.^54^

### Replication and Statistical Analysis

All experiments (poison plate, MIC, IC_50_, fungistatic activity, membrane integrity, mycelia morphology, and in vivo assays) were performed in triplicate (*n* = 3). Statistical analyses were performed using the free software R version 4.5.2. For IC_50_ estimation, a four-parameter logistic (4PL) model was used. For the other assays, Welch’s *t*-test, commonly used in biological colonization experiments, was employed to compare the treatment and control groups with potentially unequal variances, and the corresponding *p*-values were calculated.^55^

## Results and Discussion

### Fungal Strain Identification and Anti-*Fusarium* Screening

Nine culturable fungal strains were recovered from various mangrove components, including branches, roots, and leaves, at Punta Sur Ecological Park, Biosphere Reserve, Mexico (Figure 1). Identification was achieved through a combination of morphological characterization and molecular sequencing of the ITS rDNA region, with taxonomic placement confirmed via BLAST searches and maximum-likelihood analysis (Supporting Information).^56^ The endophytic fungi were identified as *Diaporthe australiana* R. G. Shiva, Akinsami & Y. P. Tan (M14 and M23); *Neofusicoccum rapaneae* W. Zhang & Crous (M21); *Cytospora heveae* Senwanna, Cheew. & K. D. Hyde (M22); *Daldinia bambusicola* Y. M. Ju, J. D. Rogers & F. San Martín (M26); *Neopestalotiopsis nebuloides* C- Lock, Vitelli, Holdom, Y. P. Tan & R. G. Shivas (M27); *Phaeoacremonium rubrigenum* W. Gams, Crous & M. J. Wing (M28); *Curvularia reesii* Y. P. Tan & R. G. Shivas (M30); and *T. islandicus* Sopp Samson, N. Yilmaz, Frisvad & Seifert (M31). These results contribute to understanding the biological diversity in unexplored areas of Mexico, such as the Punta Sur Ecological Park.

**Figure 1:**
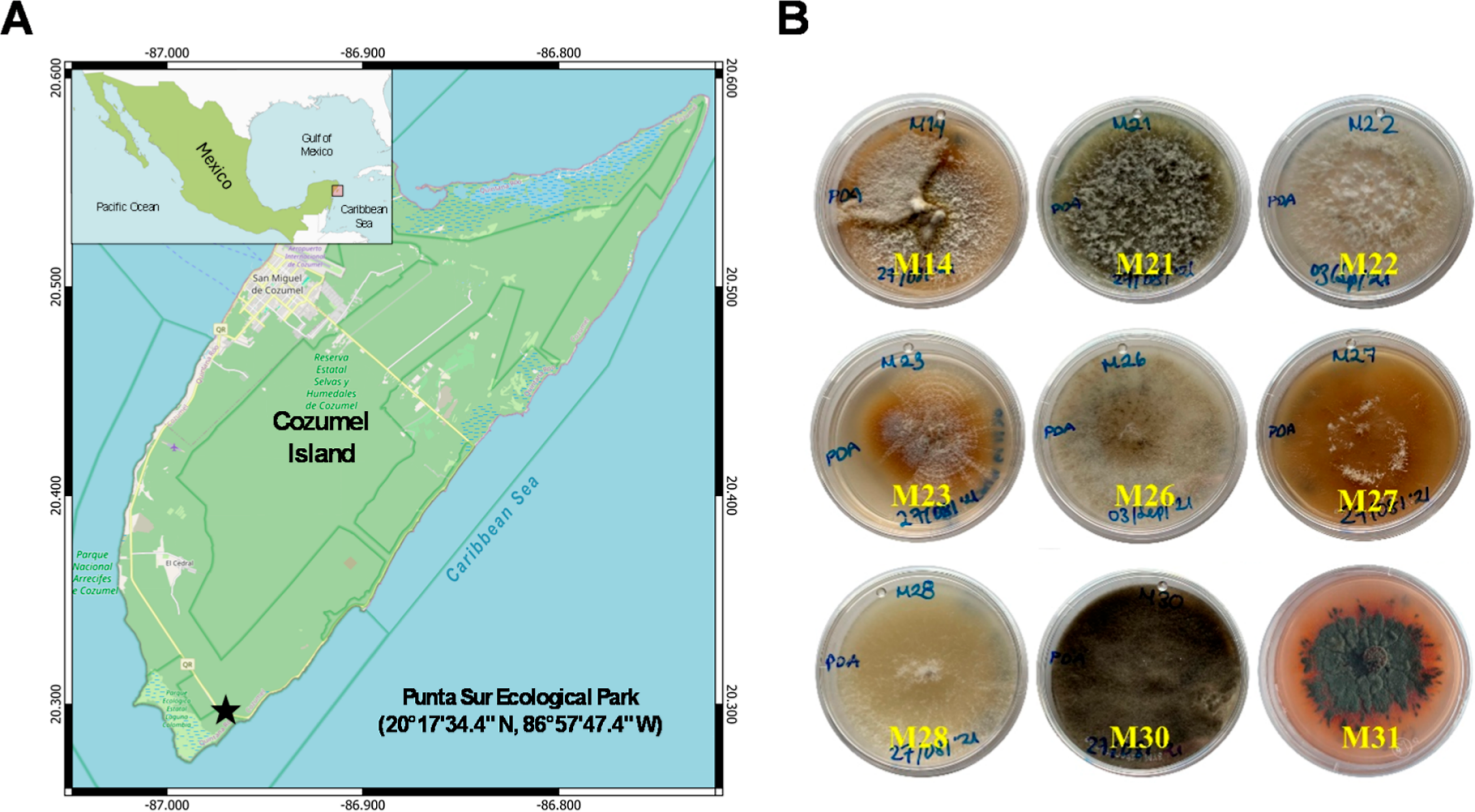
(A) Location of Punta Sur Ecological Park in Cozumel Island Biosphere Reserve, Mexico, and (B) fungal isolates from mangrove segments on PDA medium: *D. australiana* M14 and M23, *N. rapaneae* M21, *C. heveae* M22, *D. bambusicola* M26, *N. nebuloides* M27, *P. rubigenum* M28, *C. reesii* M30, and *T. islandicus* M31.

Next, the organic extract of each strain was evaluated in the poison-plate assay against the agave pathogens *F. proliferatum* CRT-142, *F. incarnatum* CRT-153, and *F. oxysporum* CRT-098, showing growth-inhibition values ranging from 20% to 74% (Supporting Information). Interestingly, the *T. islandicus* M31 was the only strain among the nine isolates to exhibit broad-spectrum inhibition against all three test pathogens (Supporting Information).

### Chemical Study and Molecular Networking Analysis of *T. islandicus* M31

The bioactive-guided chemical study of the scale-up culture (10×) of *T. islandicus* M31 yielded eight known anthraquinone derivatives identified by comparison with reported spectroscopic and spectrometric data (Supporting Information), although the NMR data of **5** have not been fully described until this report: islandicin (**1**),^57^ catenarin (**2**),^58^ (+)-iridoskyin (**3**),^59^ (+)-skyrin (**4**),^60^ (+)-aurantioskyrin (**5**),^61^ (−)-luteoskyrin (**6**), (−)-rubroskyrin (**7**), and (−)-deoxyrubroskyrin (**8**)^57^ (Figure 2). In addition, suitable red crystals of **7** were obtained for X-ray structural determination, thereby confirming its absolute configuration (Figure 1 and Supporting Information). Finally, the absolute configuration of **5** was established by comparing its ECD spectra with those of **3** and **4** (Figure 3).^57,60^

**Figure 2:**
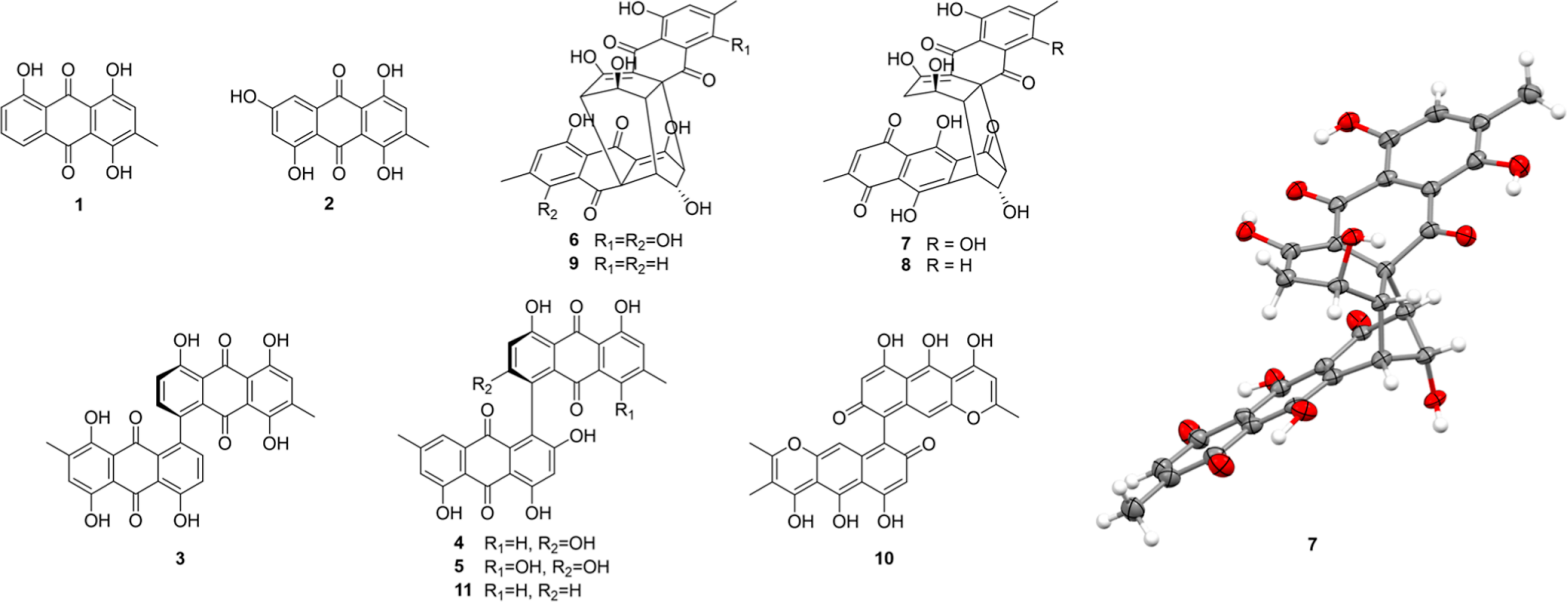
Structures of compounds **1–8** isolated from *Talaromyces islandicus* M31 and **9**–**11** identified by GNPS and displacement ellipsoid plot (50% probability level) of **7** at 100(2) K.

**Figure 3:**
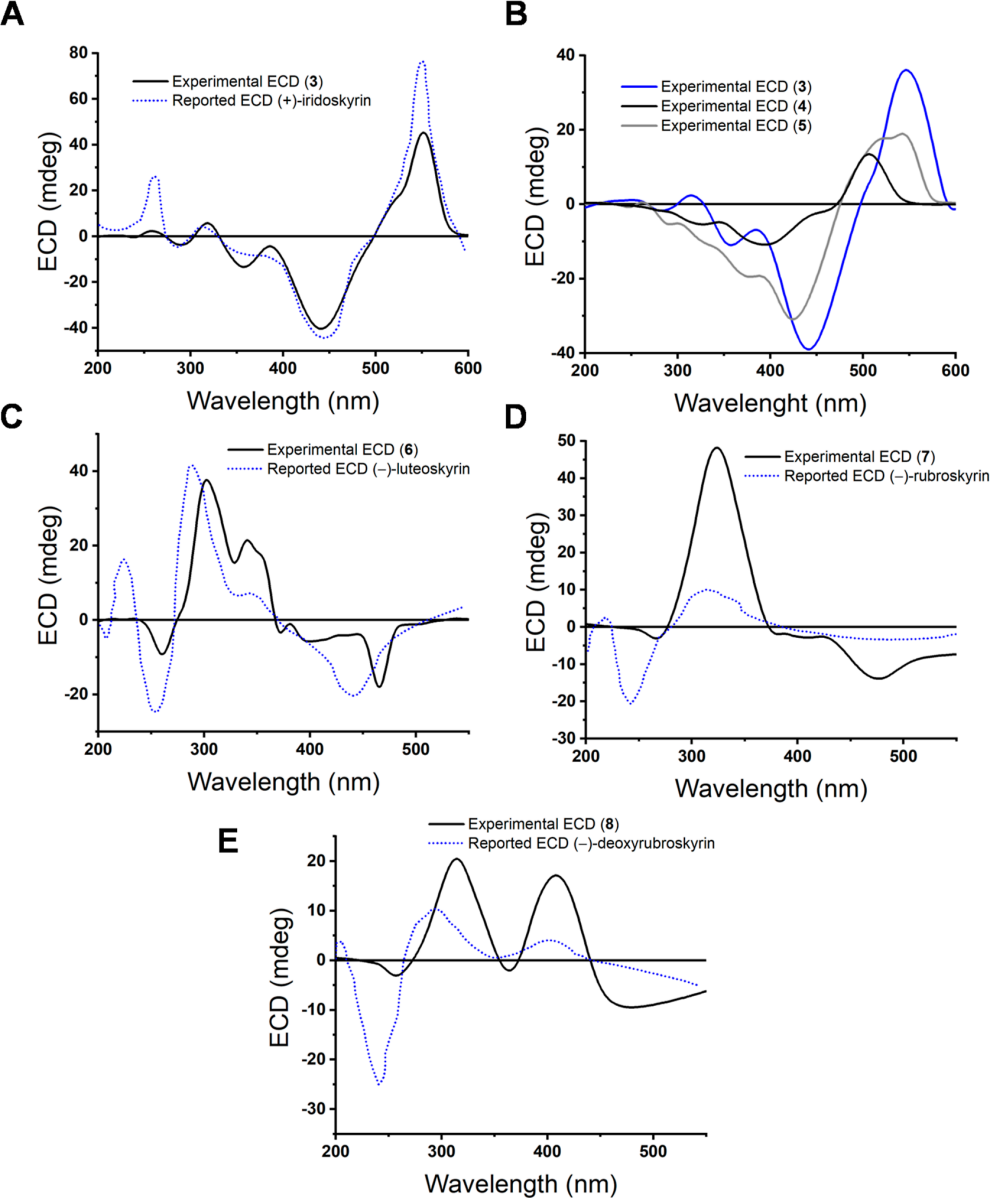
(A) Comparison of ECD spectra of (+)-iridoskyrin (**3**) (2.5 M, 1,4-dioxane) and reported^57^ and (B) between compounds **3**, (+)-skyrin (**4**) (2.5 M, 1,4-dioxane), and (+)-aurantioskyrin (**5**) (2.3 M, 1,4-dioxane). (C) Comparison of ECD spectra of (−)-luteoskyrin (**6**) (2.3 M, 1,4-dioxane), (D) (−)-rubroskyrin (**7**) (3.5 M, 1,4-dioxane), and (E) (−)-deoxyrubroskyrin (**8**) (3.6 M, 1,4-dioxane) and reported.^57^

Next, the organic extract of *T. islandicus* M31 was analyzed by UPLC-HRESIMS/MS and subjected to FBMN analysis using the GNPS2 platform (Figure 4). The resulting metabolite features were grouped into 447 nodes, organized into 32 clusters with >3 nodes per cluster, 29 with two nodes, and 110 singletons. The chemical ontology analysis cluster facilitated the manual annotation of compounds **1**–**8** since these were not annotated in the GNPS2 database, as well as the identification by SIRIUS of the additional compounds in the extract of the same chemical classes: rugulosin (**9**), ustilaginoidin P (**10**), and auroskyrin (**11**) (Figure 4 and Supporting Information).

**Figure 4:**
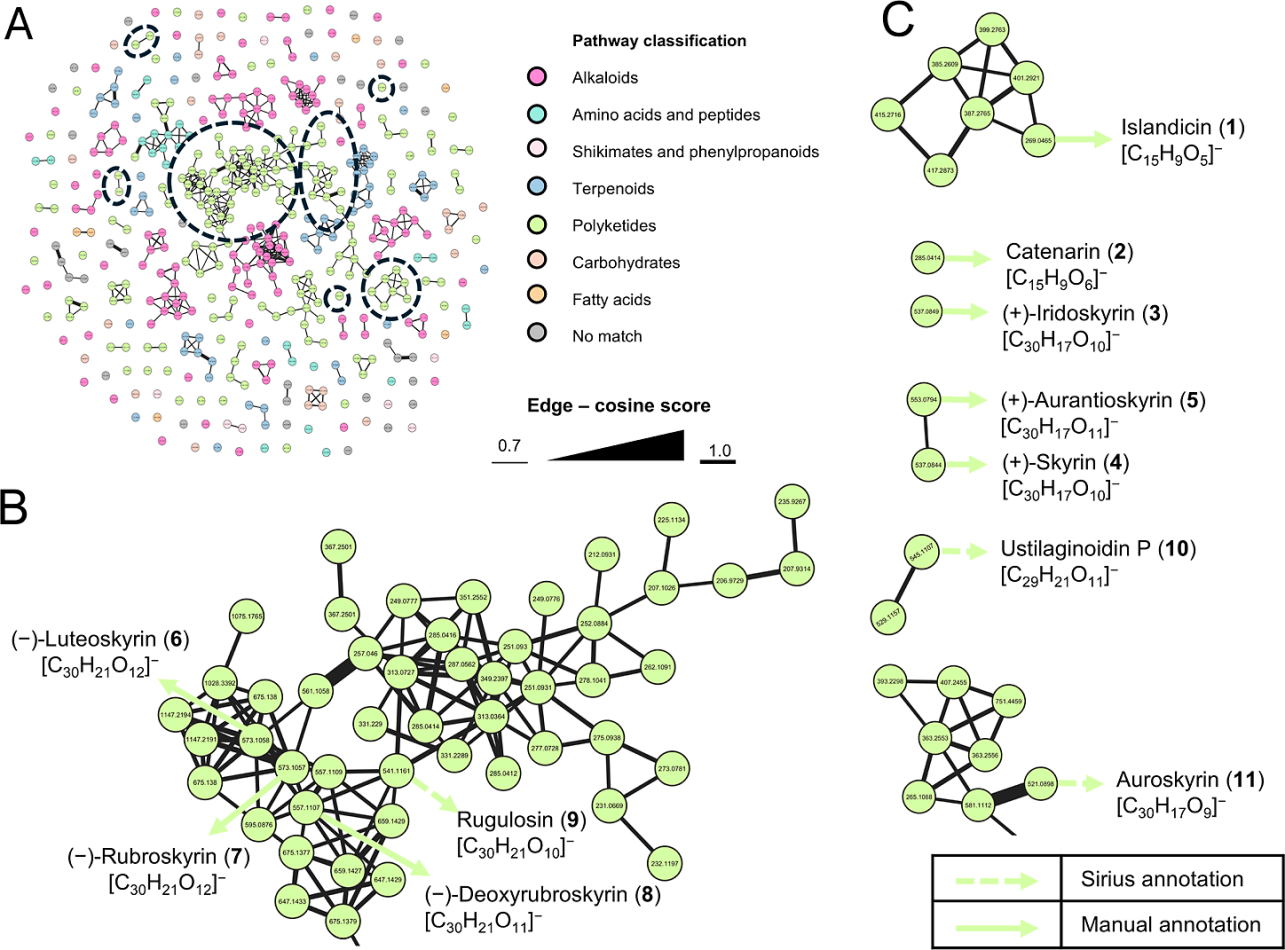
Metabolomic analysis of *T. islandicus* M31. (A) FBMN, including chemical classification, and (B,C) selected clusters and nodes showing the compounds manually annotated and by SIRIUS search.

### Biological Activity of Pure Compounds

Compounds **1–8** have previously been reported to possess antibacterial, antifungal, and phytotoxic activities (Supporting Information). In this work, compounds **1–7** were evaluated at 100 μg/mL in the poison-plate assay against *F. proliferatum* CRT-142, *F. incarnatum* CRT-153, and *F. oxysporum* CRT-098. Due to the limited availability, compound **8** was not tested in this assay. Compounds **1** and **3** showed anti-*Fusarium* activity of <35%; **2**, **4**, **5**, and **7** showed 35–70%; and **6** exhibited the highest inhibition (>83%). Based on these results, compounds **4–7** were selected for their anti-*Fusarium* activity using a broth microplate method over 96 h against all five agave pathogens (Figure 5). Compound **2** was not included in this assay due to its limited availability, whereas **8** was tested because the microdilution requires only small quantities of material. Among all, **6** showed the strongest growth inhibition against *F. incarnatum* CRT-153 and CRT-197 and *F. oxysporum* CRT-214 strains throughout the 96 h of assay (inhibition ranging from 85% to 98%), with MIC and IC_50_ values ranging from 8.7 to 34.9 μM and 4.4 to 16.0 μM, respectively (Figure 5 and Supporting Information). Finally, the long-term growth inhibition of *F. incarnatum* CRT-153 and CRT-197 by MIC and 2 × MIC concentrations of **6** was evaluated over time. For this, fungal growth on PDB was monitored, and an aliquot of the MIC or 2 × MIC of **6** was added weekly to each flask for 2 months. As observed in Figure 6, the growth of *F. incarnatum* strains remained inhibited throughout the experimental period, suggesting fungicidal activity and a new potential alternative strategy for controlling these agave pathogens in agriculture, where synthetic pesticides are traditionally applied to the plants weekly.^62^ Interestingly, this compound has shown low-to-moderate antifungal activity against other fungal plant pathogens, *Alternaria brassicicola*, *Colletotrichum acutatum*, and *Magnaporthe grisea*,^63^ but its activity against *Fusarium* species has not yet been reported.

**Figure 5:**
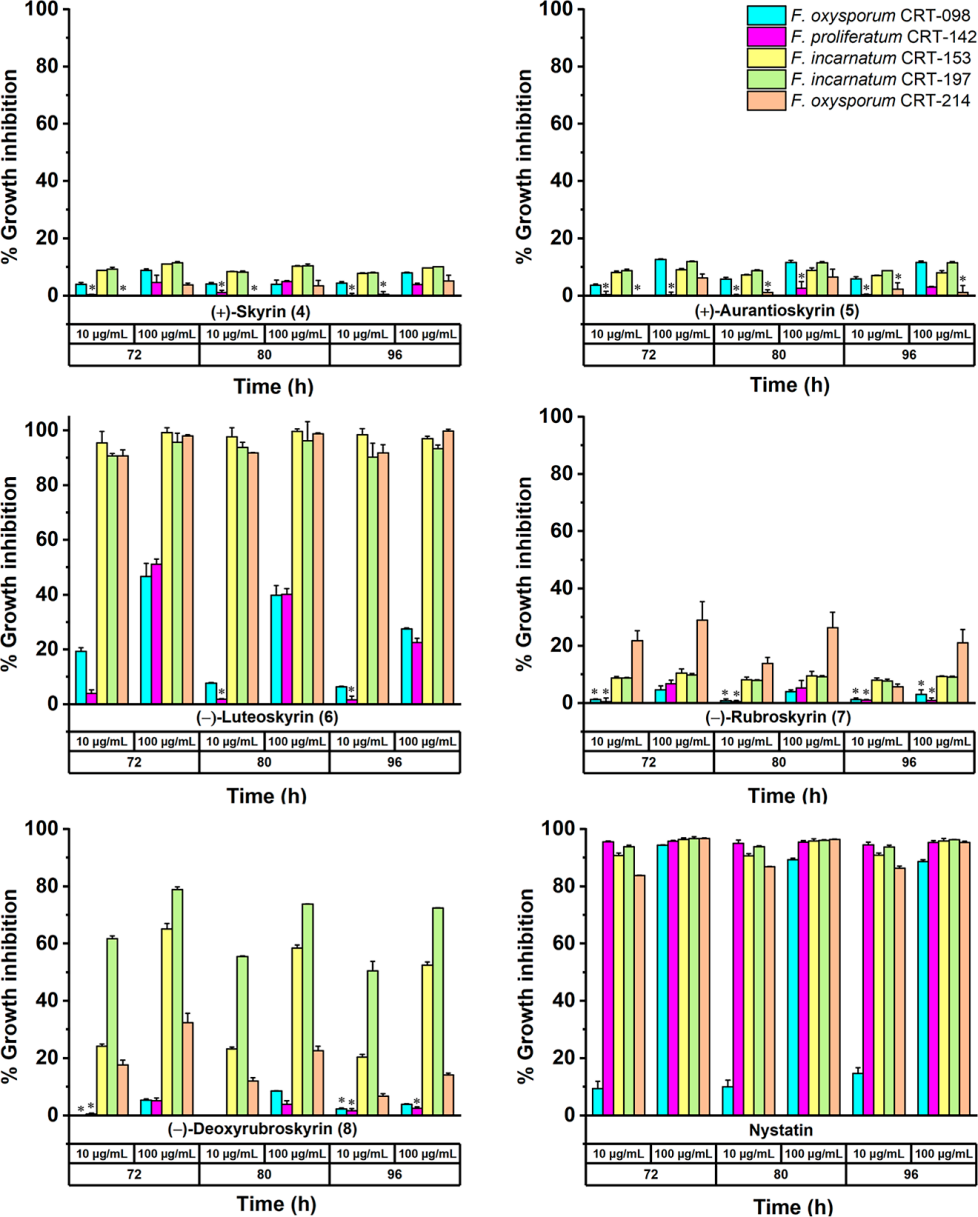
Antifungal activity of compounds **4–8** and nystatin (positive control) against *Fusarium* agave pathogens over 96 h (broth microdilution method). *No statistical significance compared to the negative control DMSO (*p* < 0.001).

**Figure 6:**
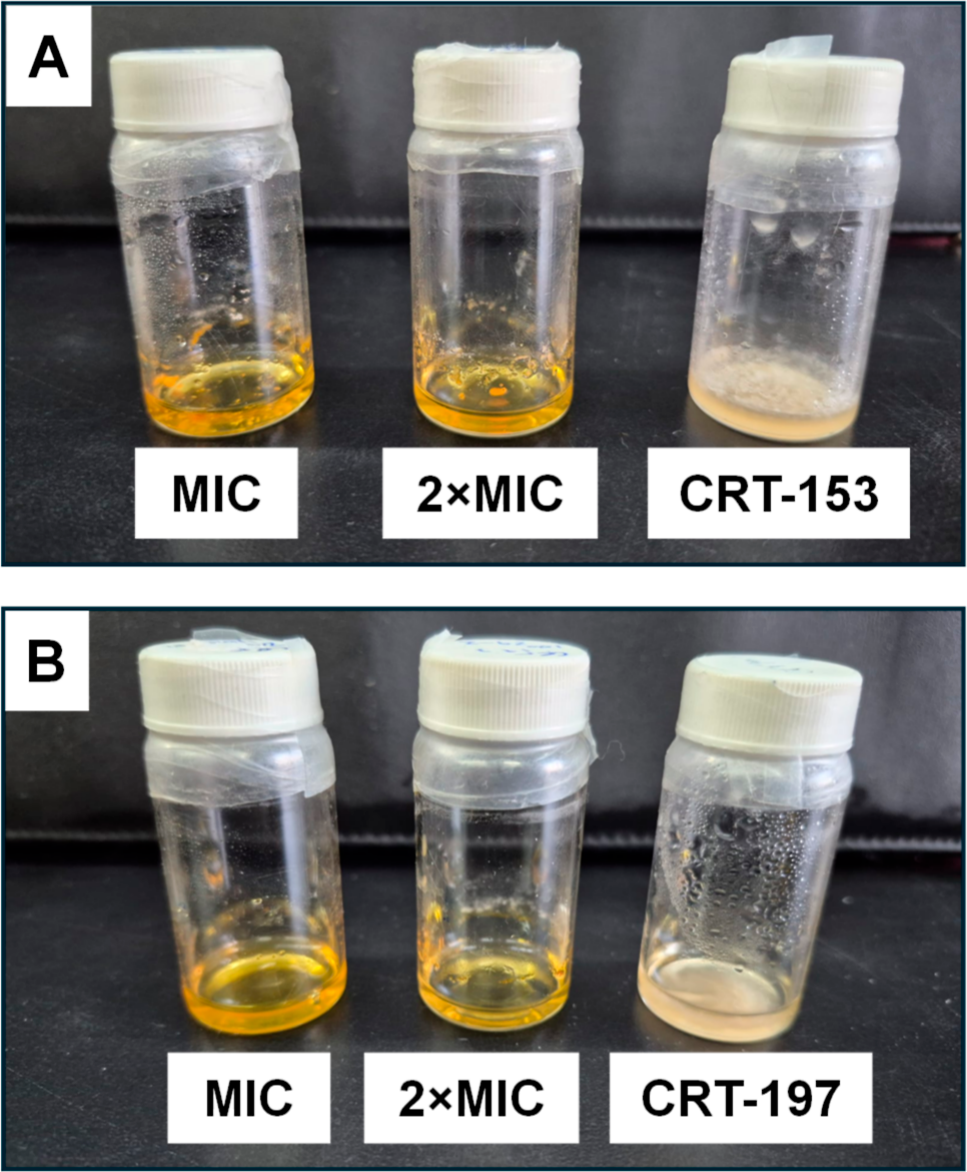
Growth inhibition of (A) *F. incarnatum* CRT-153 and (B) CRT-197 at MIC and 2 × MIC concentrations of **6** after two months of assay.

### Antifungal Mechanism of Action of (−)-Luteoskyrin (**6**)

The growth inhibition of *Fusarium* species by natural products has been studied and, in many cases, attributed to alterations at the membrane level.^64^ Thus, the antifungal action of **6** was tested by measuring the release of macromolecules (proteins and nucleic acids) from the hyphae of strains *F. incarnatum* CRT-153 and CRT-197 into the culture medium in the presence of **6** (Table 1). For strain CRT-153, we found a 7.5-fold and 19-fold increase in nucleic acids and protein content, respectively, in the extracellular media, compared with the nontreated controls. Similarly, a significant increase in macromolecular concentration was observed with nystatin, a well-known antifungal agent that binds to ergosterol in the fungal membrane, thereby increasing its permeability. *F. incarnatum* strain CRT-197 showed comparable results. The increased amount of macromolecules in the culture medium of treated samples compared to nontreated mycelia and the positive control suggests damage to the *Fusarium* cell membrane by **6**. To further investigate these effects, the mycelia were examined using SEM. As shown in Figure 7, the *F. incarnatum* CRT-197 membrane exposed to **6** revealed noticeable morphological alterations in the hyphae, specifically the shriveling and flattening of *Fusarium* hyphae, which are indicative of a profound loss of internal turgor pressure. This physical manifestation correlates strongly with our quantitative leakage data (Table 1) and aligns with the established mechanism of membrane-active antifungals, such as nystatin. For instance, the activity of (−)-luteoskyrin (**6**) mirrors that of polyene antibiotics and certain cinnamaldehyde derivatives and certain phenolic compounds,^63–65^ which compromise the selective permeability of the fungal lipid bilayer. Such disruption leads to the uncontrolled release of essential cytoplasmic constituents, such as proteins and nucleic acids, ultimately resulting in irreversible cellular collapse, as visualized in this study by SEM. However, it is unlikely that **6** acts through a single or unique mechanism.

**Table 1:** Membrane Integrity Effect in *F. incarnatum* CRT-153 and CRT-197 Hyphae by (−)-Luteoskyrin (**6**)

	CRT-153		CRT-197	
sample	protein (μg/mL)^a^	nucleic acid (ng/μL)^a^	protein (μg/mL)^a^	nucleic acid (ng/μL)^a^
(−)-luteoskyrin (**6**)	10.5 ± 2.3^b^	54.5 ± 2.6^c^	8.8 ± 1.7^c^	36.4 ± 4.0^b^
nystatin	5.7 ± 0.3^b^	85.5 ± 3.7^c^	5.1 ± 0.6^c^	78.6 ± 5.2^b^
blank media	1.4 ± 0.7	2.9 ± 0.5	1.3 ± 0.8	3.4 ± 1.2
ketoconazole	1.7 ± 0.6	11.2 ± 1.8	1.6 ± 0.7	10.7 ± 0.9

a Data are expressed as the mean ± SEM of three independent experiments. Statistical significance of protein or nucleic acid concentration compared with the control group.

b *p* < 0.05.

c *p* < 0.001.

**Figure 7:**
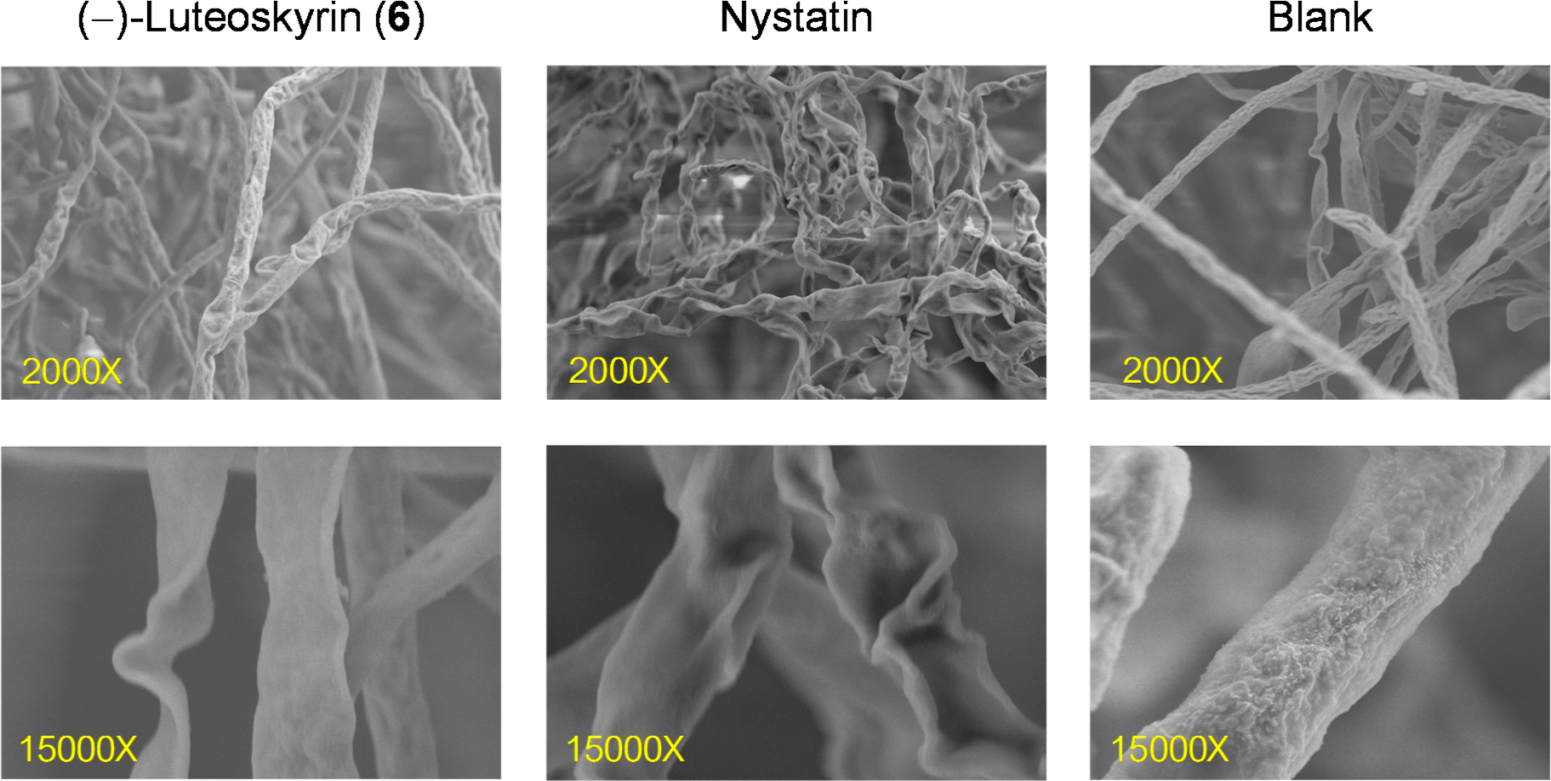
SEM morphology of *F. incarnatum* CRT-197 mycelium in the presence of **6**.

### In Vivo Activity of the Fungal Mycelial Formulation on Agave Plants Infected with *F. incarnatum* Strains

Because of the anti-*Fusarium* activity observed with the extract and the isolated compounds from *T. islandicus* M31, a simple formulation consisting of inactivated mycelium of the fungus grown in rice media was tested in vivo to control infection by *F. incarnatum* CRT-153 and CRT-197 on agave plants. To assess toxicity to the agaves, the formulation was first applied at 0.15 mg/cm^3^ and 0.75 mg/cm^3^ to the potted plant surfaces. After a month of application of the formulation, no morphological changes in the agave leaves or roots were observed (Supporting Information). Then, agave-infected plants with *F. incarnatum* CRT-153 and CRT-197 were treated with the formulation at the same concentrations, and disease severity was assessed based on physical and biological changes (Figure 8) and by quantifying pathogen colonization in agave plant roots (Figure 9). In infected plants without treatment (controls), roots showed loss of or severe damage to roots, leaves lost coloration and rigidity, and normal growth ceased (Figure 8). On the other hand, treatment with 0.15 mg/cm^3^ temporarily controlled the disease for 2 weeks, after which partial root and stem damage became apparent, necessitating a second dose of formulation (data not shown). In contrast, the 0.75 mg/cm^3^ treatment effectively controlled the infection of *F. incarnatum* CRT-153 and CRT-197 in both agave species (Figure 8). Interestingly, morphological observations of *Fusarium* infection control in agaves by the formulation were confirmed in the pathogen-colonization assay. Thus, after 96 h of incubation of agave roots on agar plates, the level of colonization of *A. angustifolia* by *F. incarnatum* CRT-153 and CRT-197 decreased by 95% and 100%, respectively, with the formulation at 0.75 mg/cm^3^. In the case of *A. potatorum*, the results were similar, with 98% and 97% reductions in colonization by *F. incarnatum* CRT-153 and CRT-197, respectively. It is important to note that while our greenhouse trials used a standardized substrate (tezontle, vermiculite, and peat moss), future field studies are required to evaluate how the formulation interacts with the native soil microbiome and the diverse soil chemistries found across Mexico’s agave-growing regions.

**Figure 8:**
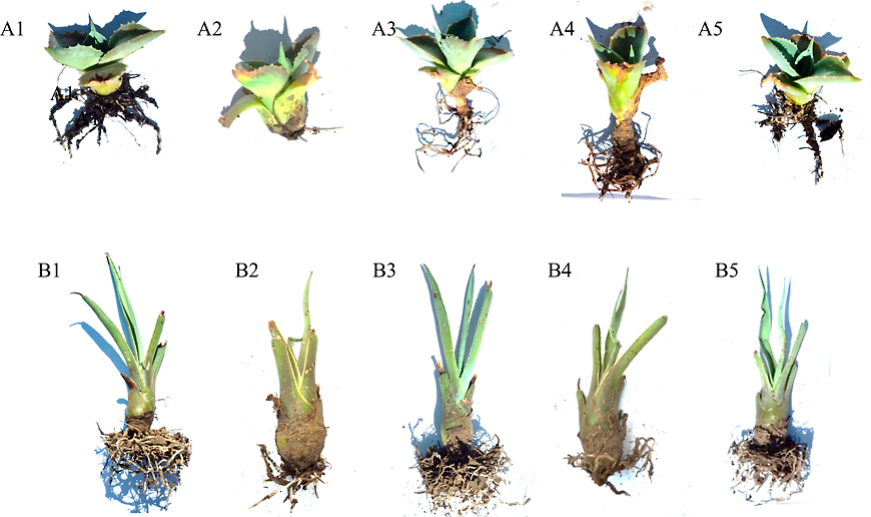
In vivo evaluation of the formulation of *T. islandicus* M31 on agave plants infected with *Fusarium* spp. Top: *A. potatorum* (A1) nontreatment and noninfection (control); (A2,A4) infected with *F. incarnatum* CRT-153 and CRT-197, respectively (disease control); and (A3,A5) infected with *F. incarnatum* CRT-153 and CRT-197 and treated with the formulation at 0.75 mg/cm^3^, respectively. Bottom: *A. angustifolia* (B1) nontreatment and noninfection (control); (B2,B4) infected with *F. incarnatum* CRT-153 and CRT-197, respectively (disease control); and (B3,B5) infected with *F. incarnatum* CRT-153 and CRT-197 and treated with the formulation at 0.75 mg/cm^3^, respectively.

**Figure 9:**
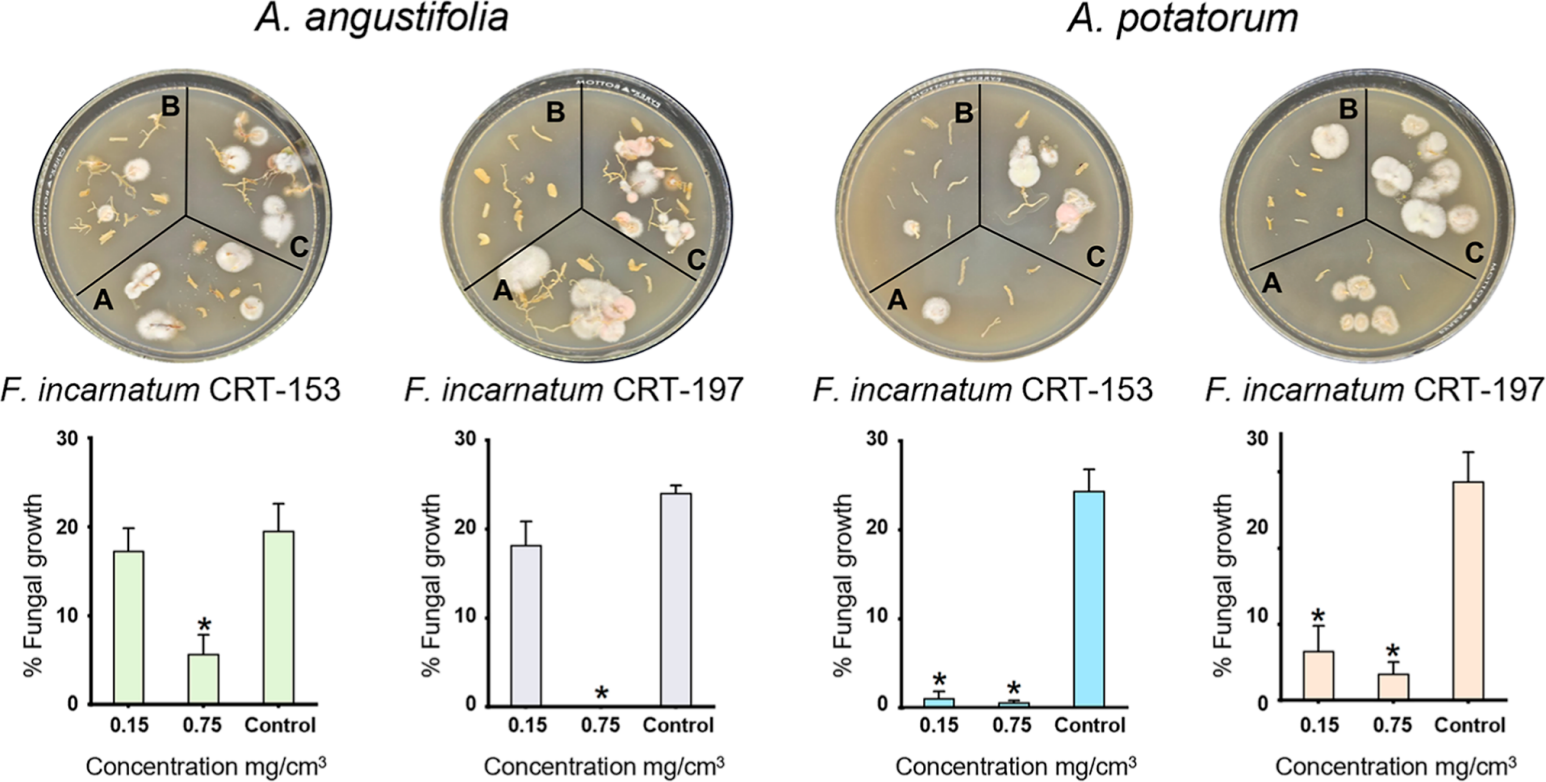
Pathogen colonization in agave plant roots. Infected (left) *A. angustifolia* and (right) *A. potatorum* agave roots treated with (A) 0.15 mg/cm^3^, (B) 0.75 mg/cm^3^, and (C) untreated (infection control). Bottom bar graphs show the percentage of growth area on the plates. The date represents the mean ± SEM of three biological replicates. *p < 0.05 (Welch’s test).

Overall, these results indicate that the simple formulation consisting of inactivated mycelium from *T. islandicus* M31 could be used as an alternative to reduce the use of synthetic fungicides for treating infected agaves and mitigate the economic impact on fermented beverages in Mexico, such as mezcal, tequila, and other distillates.^66,67^ It is worth noting that the amount of compound **6** in the fungal mycelial formulation is around 3.7% (52% in the crude extract), which is very low compared with other commercial fungal natural product-based fungicide products, such as azoxystrobin products, which contain around 23% of the active ingredient,^68^ suggesting an ability to act synergistically with other components within the fungal mycelial formulation and resulting in remarkable anti-*Fusarium* activity in vivo.

Despite the promising in vivo results of the *T. islandicus* M31 formulation, several factors must be addressed to transition this biocontrol strategy to commercial-scale agriculture. First, regarding scalability, the use of solid-state fermentation on rice, while effective for laboratory trials, requires optimization for mass production.^69^ Future efforts should evaluate agricultural byproducts (such as corn stover or agave bagasse) as lower-cost substrates to enhance the formulation’s economic viability. Second, the regulatory landscape for inactivated microbial products (organic pesticides) in Mexico, governed by the National Service for Agrifood Health, Safety, and Quality (SENASICA),^70^ remains a critical hurdle but is easier than that for synthetic pesticides or live biopesticides. An inactivated mycelium functions as a complex mixture of bioactive metabolites with environmentally friendly properties (biodegradability and biocompatibility). However, characterizing the long-term stability and standardized dosage of these compounds is essential for legal registration and environmental safety certification. Finally, to ensure broad efficacy, future field trials must move beyond controlled greenhouse settings to account for environmental variability. This includes testing the formulation across the diverse pedological conditions of Mexico’s “Denomination of Origin” regions, specifically comparing its performance in the mineral-rich volcanic soils of the Tequila region in Jalisco and Nayarit against the calcareous, coastal soils of the Oaxacan mezcal regions. Such ongoing studies will clarify how interactions within the soil microbiome and geochemical factors influence the degradation, persistence, and bioactivity of the anthraquinones.

## Supplementary Material



## Notes

Phylogenetic analysis, list of isolated pathogens, spectroscopic and spectrometric data of **1–8** [the NMR data for compounds **1–8** have been deposited in the Natural Products Magnetic Resonance Database (np-mrd.org) under accession numbers np0259613 (compound **1**), np0352058 (compound **2**), np0004754 (compound **3**), np0352059 (compound **4**), np0352060 (compound **5**), np0281378 (compound **6**), np0352061 (compound **7**), and np0085464 (compound **8**)], and in vitro and in vivo biological assay data (PDF)
