# Supplementary material for: Sustainable Biocontrol of Agave Vascular Wilt Using an Inactivated Mycelial Formulation from the Mangrove Endophyte Talaromyces islandicus M31
Source: ACS Omega. 2026 Apr 21;11(17):25849–60. doi: 10.1021/acsomega.6c01311 (PMC13150580; doi:10.1021/acsomega.6c01311)
Supplement: Supplementary file 1 [file ao6c01311_si_001.pdf]

Supporting information

Sustainable biocontrol of agave vascular wilt using an inactivated mycelial formulation from the mangrove endophyte *Talaromyces islandicus* M31

Albert D. Patiño, Javier Plasencia, Sandip Das, Shabnam Hematian, Tania Raymundo, Carmina Montiel, Ricardo Valenzuela, and Mario Figueroa*
*Corresponding Author: E-mail: mafiguer@unam.mx

Table of Contents

	Page
**Phylogenetic Affinities of Mangrove Endophytes from Punta Sur Ecological Park in Cozumel Island, Mexico.**	S3
**Figure S1.** Maximum likelihood phylogeny tree of fungal endophytes based on the ITS sequence alignment.	S3
**Figure S2.** *Fusarium* strains isolated from *A. tequilana* Weber var. *azul*-infected plantation in Jalisco, Mexico.	S4
**Table S1.** Location of the agave-infected plantations for the isolation of the *Fusarium* pathogens.	S4
**Figure S3.** Crystals of (−)-rubroskyrin (**7**).	S4
**Table S2.** Crystallographic data and data collection parameters for (−)-rubroskyrin (**7**).	S5
**Table S3.** Anti-*Fusarium* activity of fungal organic extracts at 200 μg/mL in the poison plate assay.	S5
**Figure S4.** Agave pathogens grow over time.	S6
**Figure S5.** ^{1}H (top) and ^{13}C (bottom) NMR spectra of islandicin (**1**) in $CDCl_3$ (400 and 100 MHz, respectively).	S7
**Figure S6.** HRESIMS negative mode (top) and UV (bottom) spectra of islandicin (**1**).	S8
**Figure S7.** ^{1}H (top) and ^{13}C (bottom) NMR spectra of catenarin (**2**) in DMSO-d_6 (600 and 150 MHz, respectively).	S9
**Figure S8.** HRESIMS negative mode (top) and UV (bottom) spectra of catenarin (**2**).	S10
**Figure S9.** ^{1}H (top) and ^{13}C (bottom) NMR spectra of (+)-iridoskyrin (**3**) in $CDCl_3$ (400 and 100 MHz, respectively).	S11
**Figure S10.** HRESIMS negative mode (top) and UV (bottom) spectra of (+)-iridoskyrin (**3**).	S12
**Figure S11**. ^{1}H (top) and ^{13}C (bottom) NMR spectra of (+)-skyrin (**4**) in DMSO-d_6 (600 and 150 MHz, respectively).	S13
**Figure S12.** HRESIMS negative mode (top) and UV (bottom) spectra of (+)-skyrin (**4**).	S14
**Figure S13.** ^{1}H (top) and ^{13}C (bottom) NMR spectra of (+)-aurantioskyrin (**5**) in acetone-d_6 (400 and 100 MHz, respectively).	S15
**Figure S14.** HSCQ spectrum of (+)-aurantioskyrin (**5**) in acetone-d_6 (400 and 100 MHz)	S16
**Figure S15.** HMBC spectrum of (+)-aurantioskyrin (**5**) in acetone-d_6 (400 MHz)	S16
**Figure S16.** COSY spectrum of (+)-aurantioskyrin (**5**) in acetone-d_6 (400 MHz)	S17
**Figure S17.** NOESY spectrum of (+)-aurantioskyrin (**5**) in acetone-d_6 (400 MHz)	S17
**Figure S18.** HRESIMS negative mode (top) and UV (bottom) spectra of (+)-aurantioskyrin (**5**).	S18
**Figure S19.** ^{1}H (top) and ^{13}C (bottom) NMR spectra of (−)-luteoskyrin (**6**) in DMSO-d_6 (600 and 150 MHz, respectively).	S19
**Figure S20.** HRESIMS negative mode (top) and UV (bottom) spectra of (−)-luteoskyrin (**6**).	S20
**Figure S21.** ^{1}H (top) and ^{13}C (bottom) NMR spectra of (−)-rubroskyrin (**7**) in acetone-d_6 (600 and 150 MHz, respectively).	S21
**Figure S22.** HRESIMS negative mode (top) and UV (bottom) spectra of (−)-rubroskyrin (**7**).	S22
**Figure S23.** ^{1}H (top) and ^{13}C (bottom) NMR spectra of (−)-deoxyrubroskyrin (**8**) in acetone-d_6 (600 and 150 MHz, respectively).	S23
**Figure S24.** HRESIMS negative mode (top) and UV (bottom) spectra of (−)-deoxyrubroskyrin (**8**).	S24
**Table S4.** Spectroscopic and spectrometric data of isolated compounds **1−8**.	S25

Table S5. GNPS metabolomics annotation in *T. islandicus* M31. S26
Table S6. Biological activity reported for compounds **1-8**. S27
Figure S25. Growth inhibition of Fusarium *species* by (–)-luteoskyrin (**6**). S27
Table S7. MIC and IC_{50} values of (–)-luteoskyrin (**6**) against agave pathogens. S28
Figure S26. (A) Fungal mycelial formulation of inactivated mycelia of *T. islandicus* M31. (B) Sterility test of the formulation after 7 days of incubation at 30°C and 12/12 h light-dark cycle. S28
Figure S27. Toxicity test of the fungal mycelial formulation of *T. islandicus* M31 in *A. potatorum* and *A. angustifolia*. S28
Figure S28. Relative quantification of **6** ion the fungal mycelial formulation of *T. islandicus* M31. S29

Phylogenetic Affinities of Mangrove Endophytes from Punta Sur Ecological Park in Cozumel Island, Mexico

Endophytic fungi ITS rDNA sequence analysis revealed diverse taxonomic affinities among the isolates (Figure S1). In total, nine ascomycetes were obtained: *Neofusicoccum* (n=1) (PV652925), *Talaromyces* (n=1) (PV652932), *Curvularia* (n=1) (PV652931), *Phaeoacremonium* (n=1) (PV652930), *Diaporthe* (n=2) (PV652924, PV652927), *Cytospora* (n=1) (PV652926), *Neopestalotiopsis* (n=1) (PV652929), and *Daldinia* (n=1) (PV652928).

The aligned nrITS dataset comprised 675 characters (including gaps), of which 497 were conserved, 157 were variable, and 134 were parsimony-informative. The three phylogenetic analyses, MP, ML, and BI, of the nrITS dataset recovered similar topologies (**Figure S1**). No significant conflict (bootstrap value >70%) was detected among the topologies obtained via the separate phylogenetic analyses. The parsimony analysis of the alignment found 852 trees of 123 steps (CI=0.2940, HI=0.1641, RI=0.4215, RC=0.5901). The best RAxML tree with a final likelihood of –52134.057245 is presented. The matrix contained 1012 distinct alignment patterns, with 5.41% of characters undetermined (gaps). Estimated base frequencies were as follows: A= 0.105378, C= 0.208013, G= 0.128314, T= 0.280322; substitution rates AC= 1.080421, AG= 1.803477, AT= 1.083422, CG= 1.003401, CT= 5.072468, GT= 1.000000; gamma distribution shape parameter α= 0.004285. In the Bayesian analysis, the standard deviation between the chains stabilized at 0.00002 after 4 million generations. No significant changes in the tree topology trace or in the cumulative split frequencies of selected nodes were observed after approximately 0.25 million generations, which were discarded as 25% burn-in.

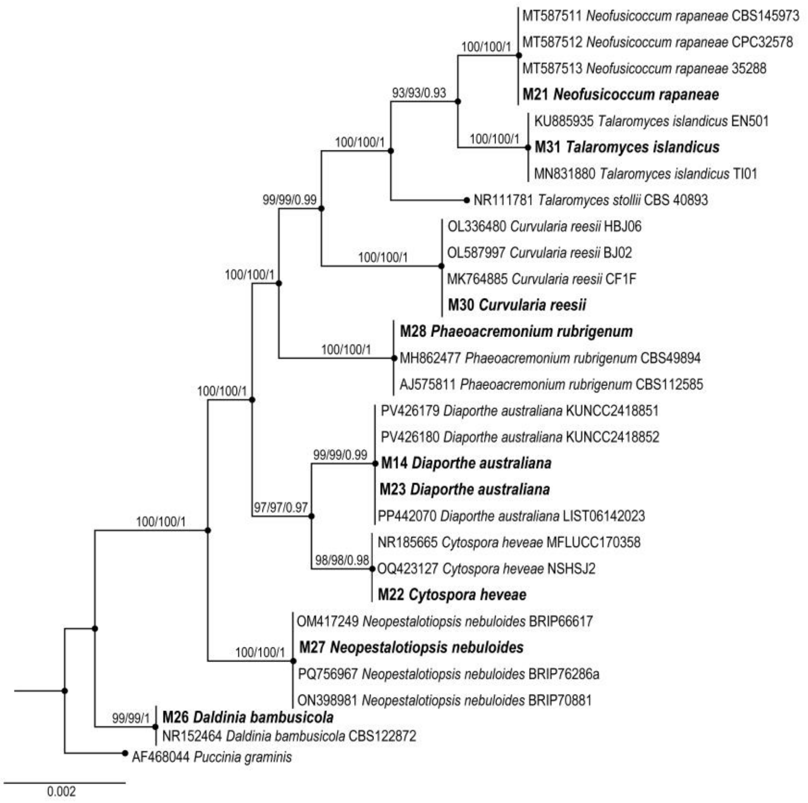

Figure S1. Maximum likelihood phylogeny tree of fungal endophytes based on the ITS sequence alignment. Maximum parsimony and Bayesian analyses recovered identical topologies. For each node, the following values are provided: maximum parsimony bootstrap (%) / maximum likelihood bootstrap (%) and posterior confidence (*p*-value). The scale bar represents the expected number of nucleotide substitutions per site.

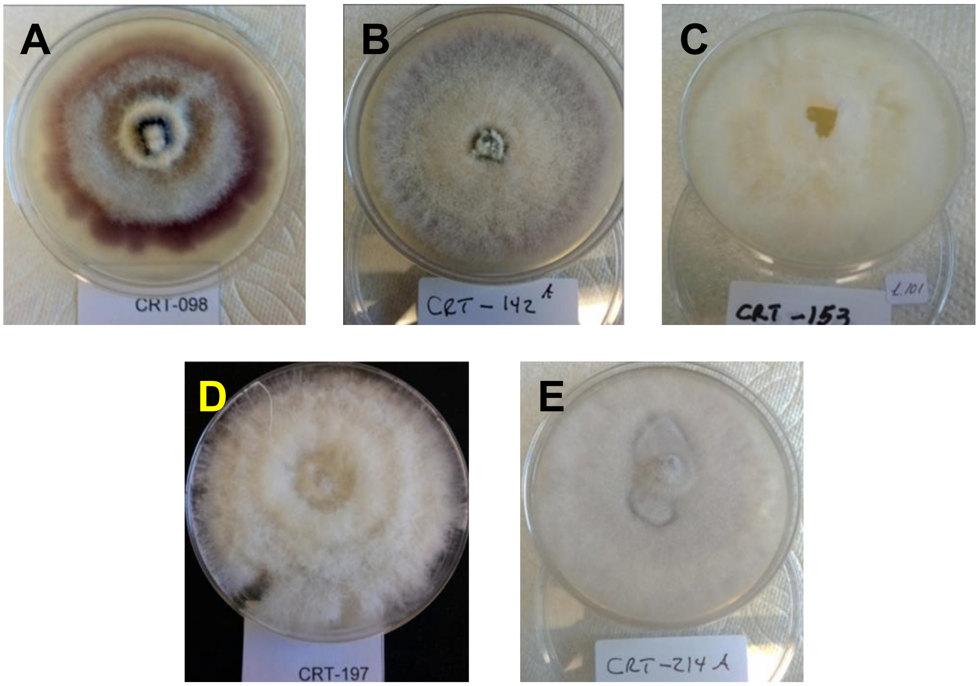

Figure S2. *Fusarium* strains isolated from *A. tequilana* Weber var. azul-infected plantation in Jalisco, Mexico. (A) *F. oxysporum* CRT-098. (B) *F. proliferatum* CRT-142. (C) *F. incarnatum* CRT-153. (D) *F. incarnatum* CRT-197. (E) *F. Oxysporum* CRT-214.

Table S1. Location of the agave-infected plantations for the isolation of the *Fusarium* pathogens.

**Code of *Fusarium***	**Latitude**	**Longitude**	**Town**	**State**
CRT-098	20.66	−102.12	Jesús María	Jalisco
CRT-142	20.65	−102.46	Atotonilco El Alto	Jalisco
CRT-153	20.65	−102.40	Arandas	Jalisco
CRT-197	20.68	−102.12	Jesús María	Jalisco
CRT-214	20.70	−102.69	Tepatitlán de Morelos	Jalisco

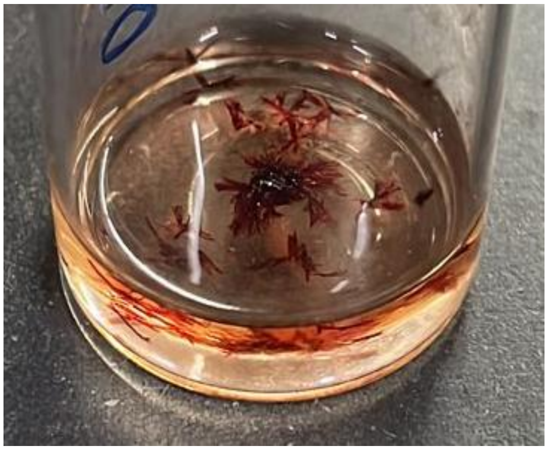

Figure S3. Crystals of (−)-rubroskyrin (**7**).

Table S2. Crystallographic data and data collection parameters for (−)-rubroskyrin (**7**).

Formula	$C_{30}H_{22}O_{12}$
Temperature/K	100.00
Formula weight	574.47
Crystal system	orthorhombic
Space group	*P* $2_1P2_1P2_1$
a/Å	7.3240(3)
b/Å	14.9535(6)
c/Å	22.2953(8)
α/°	90
β/°	90
γ/°	90
Volume/Å^3	2441.77(17)
Z	4
Radiation	CuKa (λ = 1.54178)
ρ_{calc}g/cm^3	1.563
F (000)	1192.0
Crystal size/mm^3	0.18 × 0.14 × 0.09
2Θ range for data collection/°	7.118 to 133.736
μ/mm^{-1}	1.042
Reflections collected	47121
Independent reflections	4329 [R_{int} = 0.1073, R_{sigma} = 0.0461]
Completeness to theta	1.74/1.00
No. of restraints	0
No. of params. refined	393
Goodness-of-fit on F^2	1.054
Final R indexes [I>=2σ (I)]	R_1 = 0.0422, wR_2 = 0.1009
Final R indexes [all data]	R_1 = 0.0506, wR_2 = 0.1062
Largest diff. peak/hole / e Å^{-3}	0.25/-0.26
Flack parameter	0.1(3)

Table S3. Anti-*Fusarium* activity of fungal organic extracts at 200 µg/mL in the poison plate assay.

Fungus	% Radial growth inhibition		
	F. proliferatum **CRT-142**	***F. incarnatum*** **CRT-153**	***F. oxysporum*** **CRT-098**
D. australiana M14	NAD	37.6±6.7	NAD
N. rapaneae M21	74.0±6.7	NAD	NAD
C. heveae M22	36.0±5.6	32.4±7.8	NAD
D. australiana M23	NAD	NAD	NAD
D. bambusicola M26	38.5±6.8	32.9±7.2	NAD
N. nebuloides M27	NAD	NAD	NAD
P. rubrigenum M28	NAD	NAD	NAD
C. reesii M30	NAD	23.4±5.6	NAD
T. islandicus M31	45.1±3.4	52.2±3.7	21.5±5.2
Ketoconazole[b]	94.1	94.7	91.0

[a]Data are shown as mean ± SEM of three biological replicates; [b]Positive control at 200 µg/mL; NAD: No activity detected.

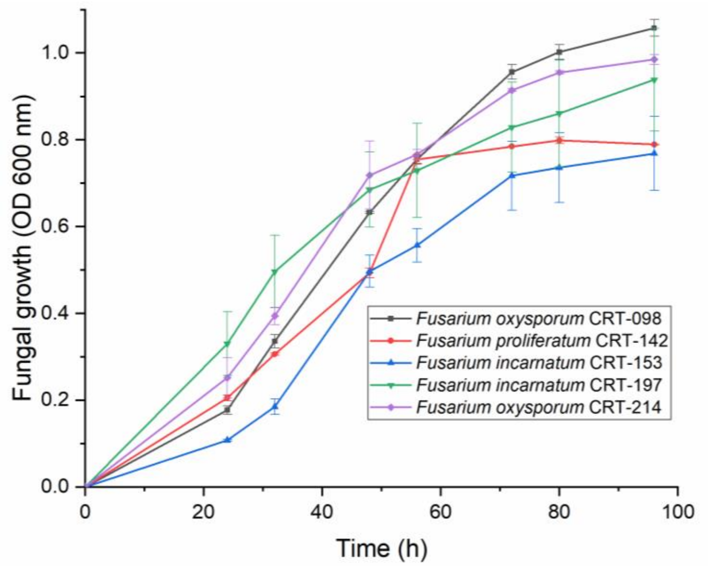

Figure S4. Agave pathogens grow over time. The data shown are the mean ± SEM from three replicates.

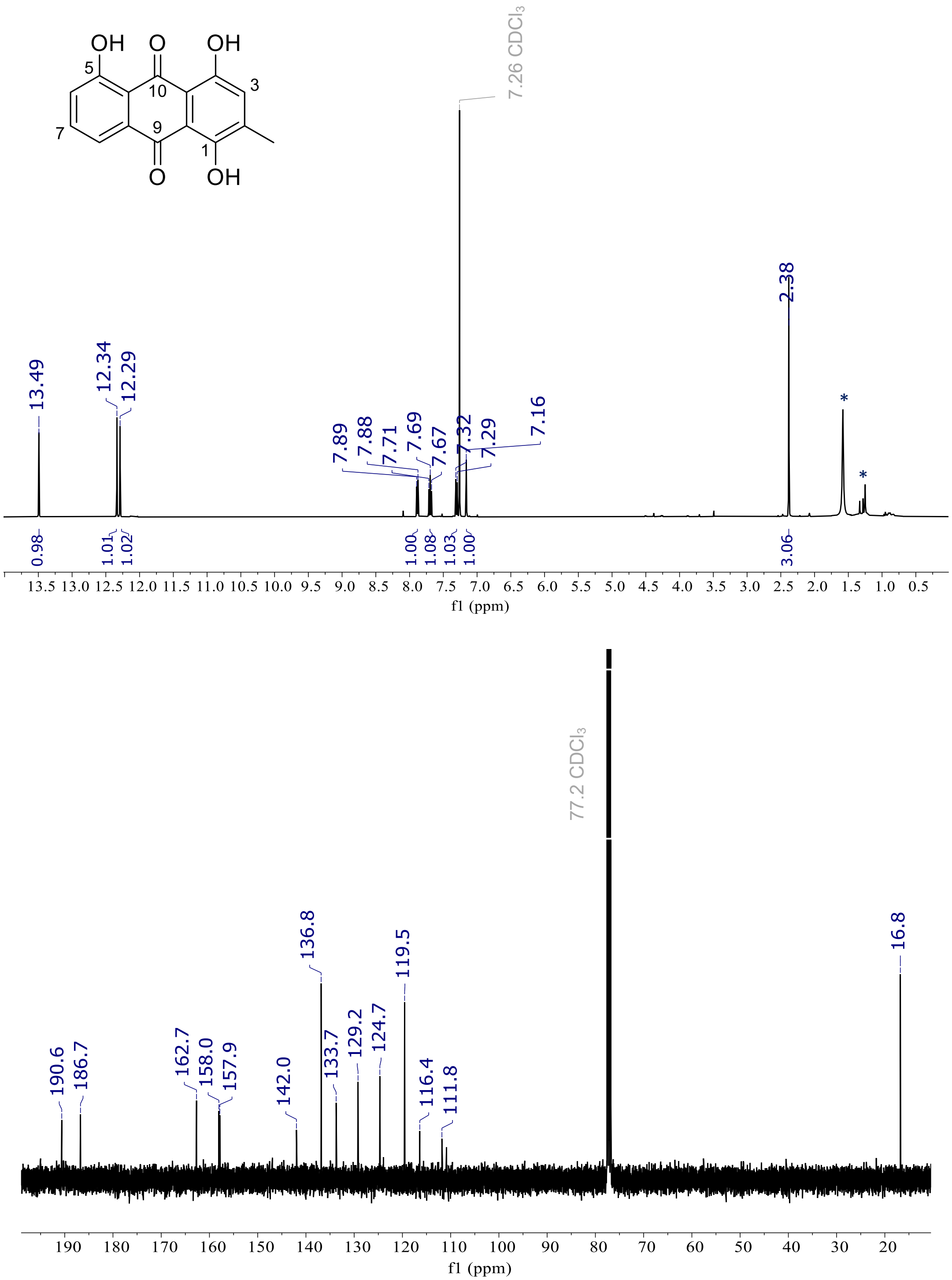

Figure S5. ^{1}H (top) and ^{13}C (bottom) NMR spectra of islandicin (**1**) in $CDCl_3$ (400 and 100 MHz, respectively). *Impurities.

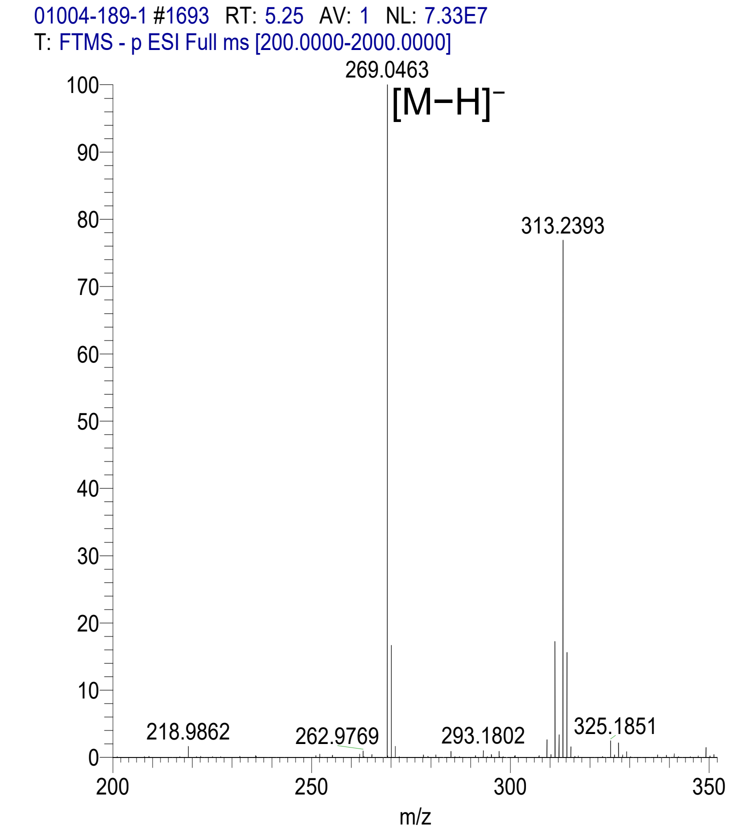

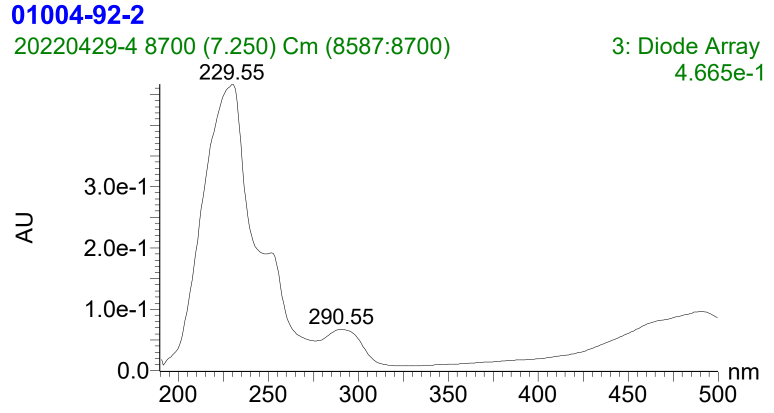

Figure S6. HRESIMS negative mode (top) and UV (bottom) spectra of islandicin (**1**).

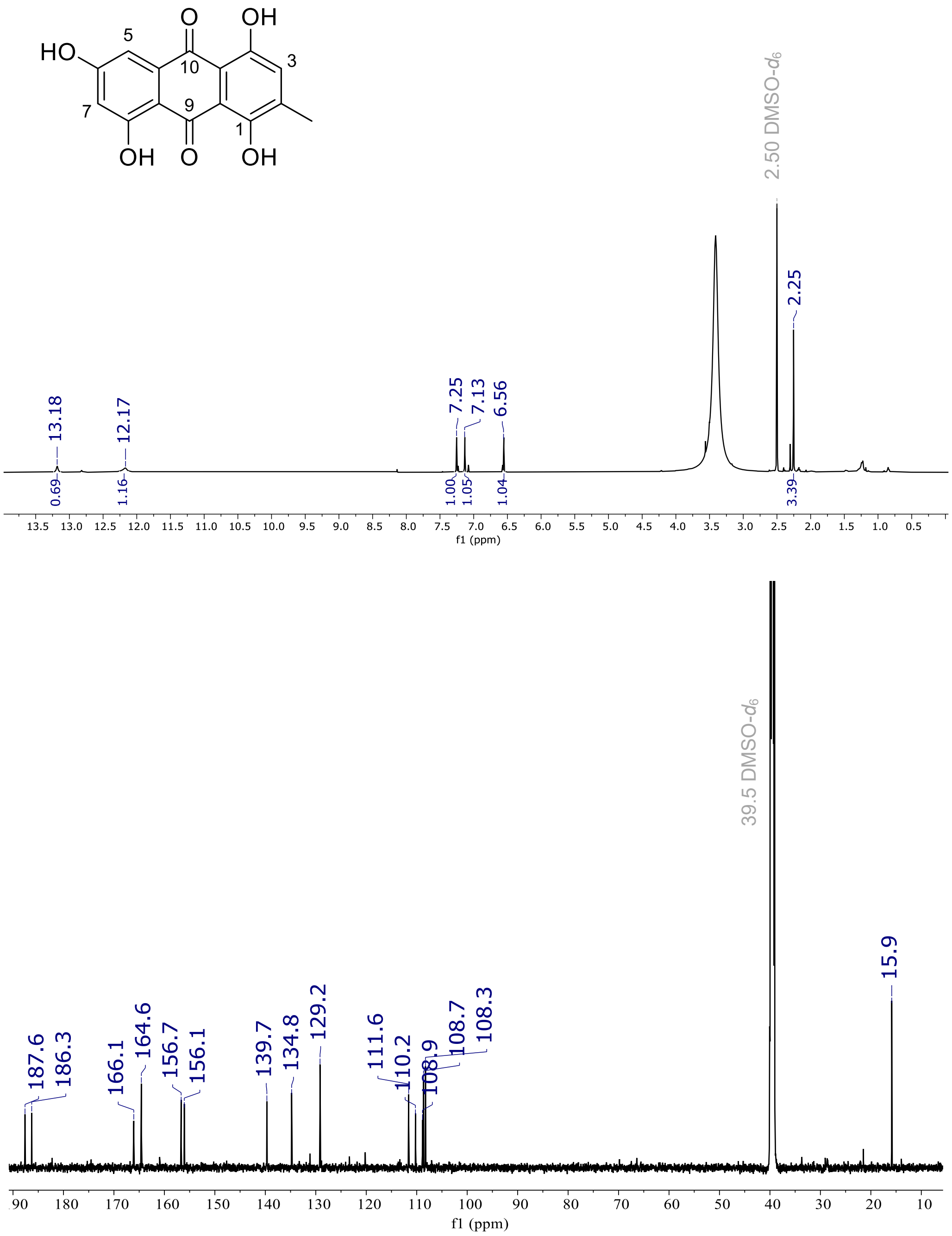

Figure S7. ^{1}H (top) and ^{13}C (bottom) NMR spectra of catenarin (**2**) in DMSO-d_6 (600 and 150 MHz, respectively).

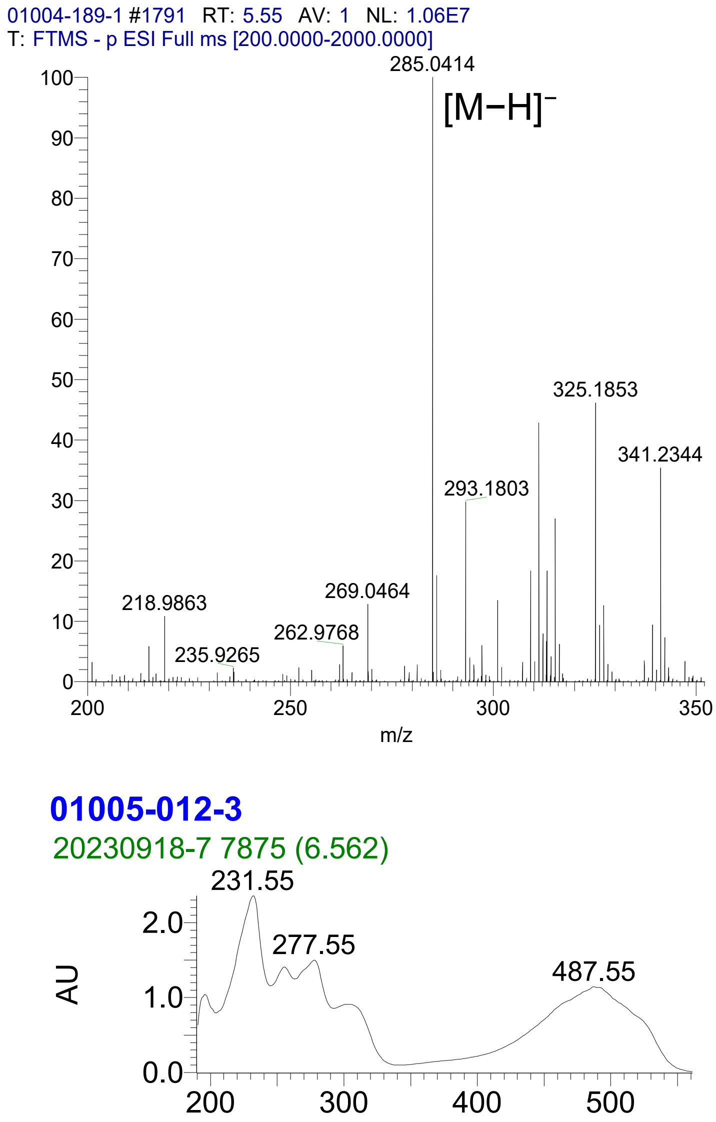

Figure S8. HRESIMS negative mode (top) and UV (bottom) spectra of catenarin (**2**).

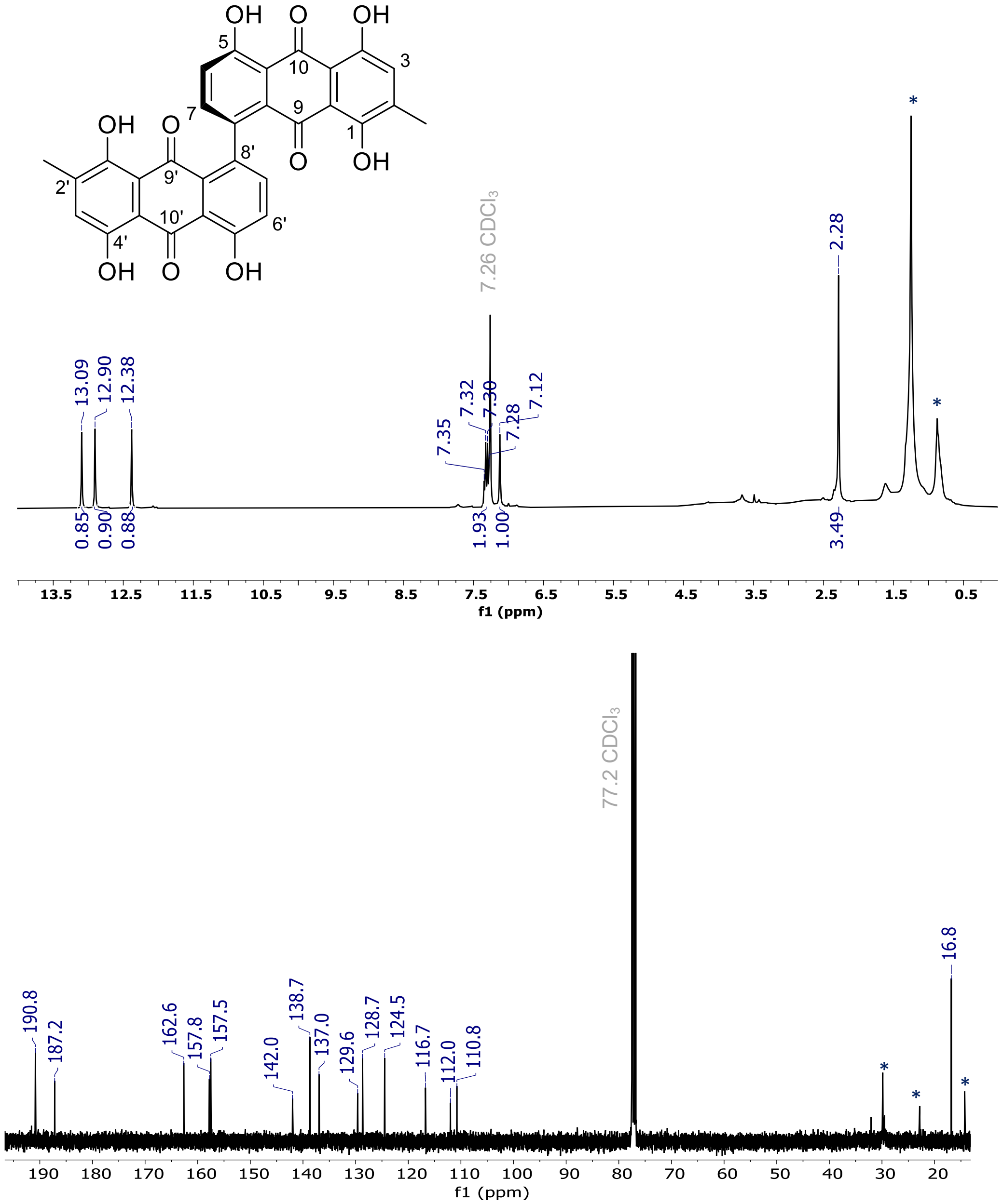

Figure S9. ^{1}H (top) and ^{13}C (bottom) NMR spectra of (+)-iridoskyrin (**3**) in $CDCl_3$ (400 and 100 MHz, respectively). *Impurities.

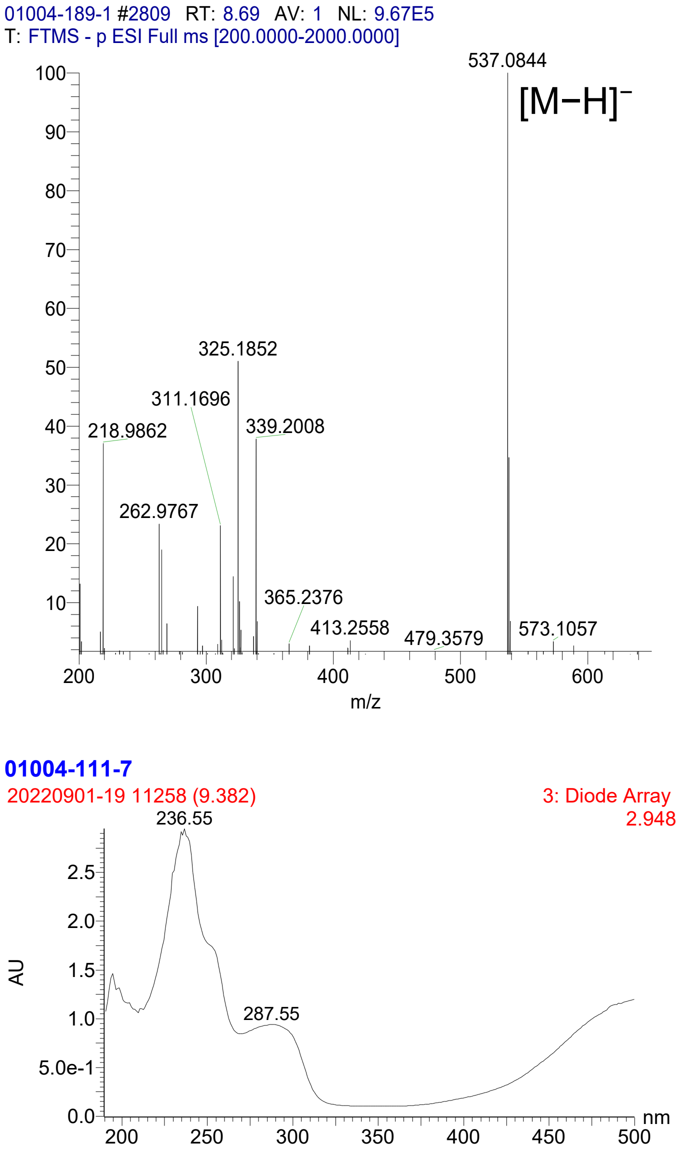

Figure S10. HRESIMS negative mode (top) and UV (bottom) spectra of (+)-iridoskyrin (**3**).

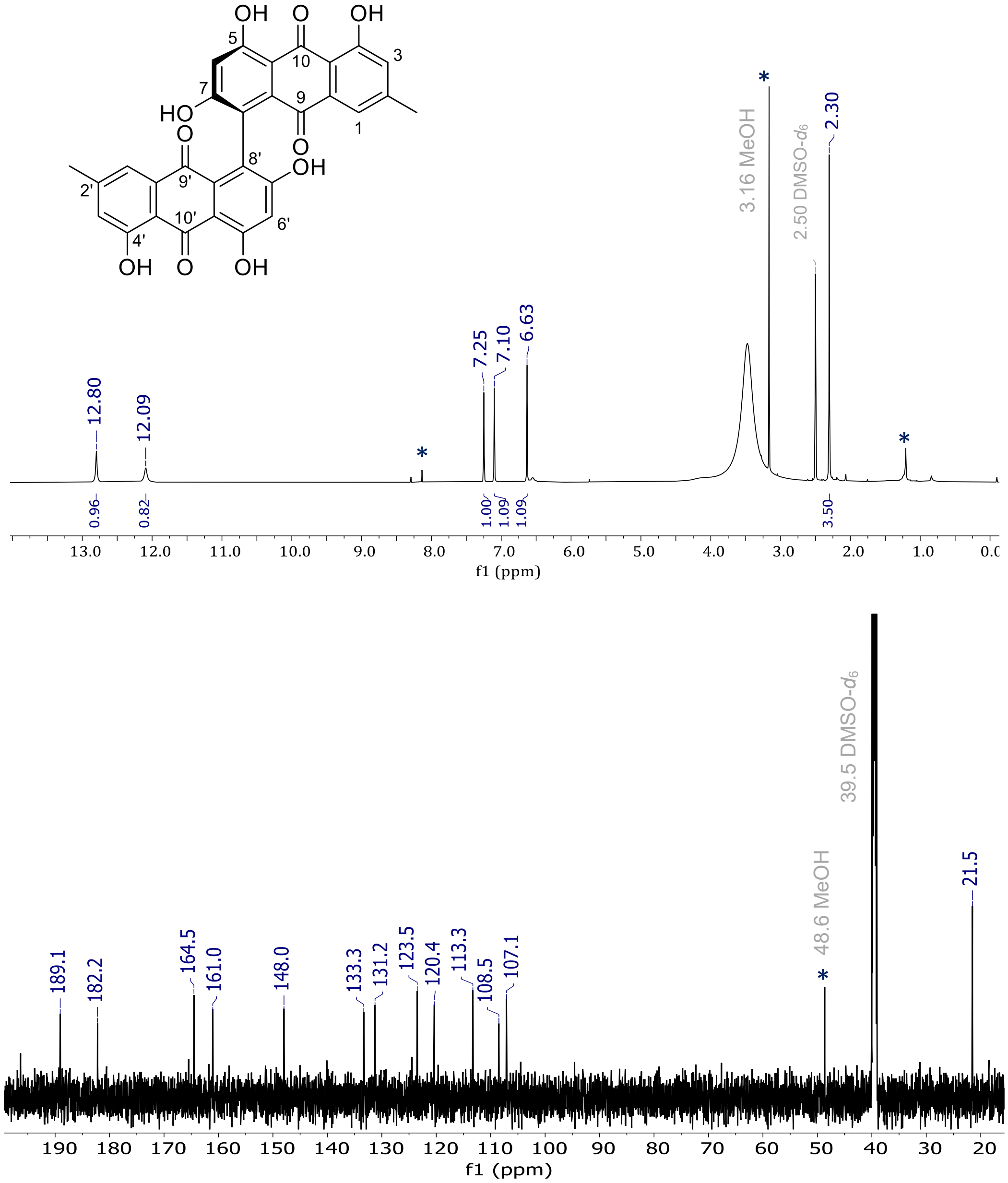

Figure S11. ^{1}H (top) and ^{13}C (bottom) NMR spectra of (+)-skyrin (**4**) in DMSO-d_6 (600 and 150 MHz, respectively). *Impurities.

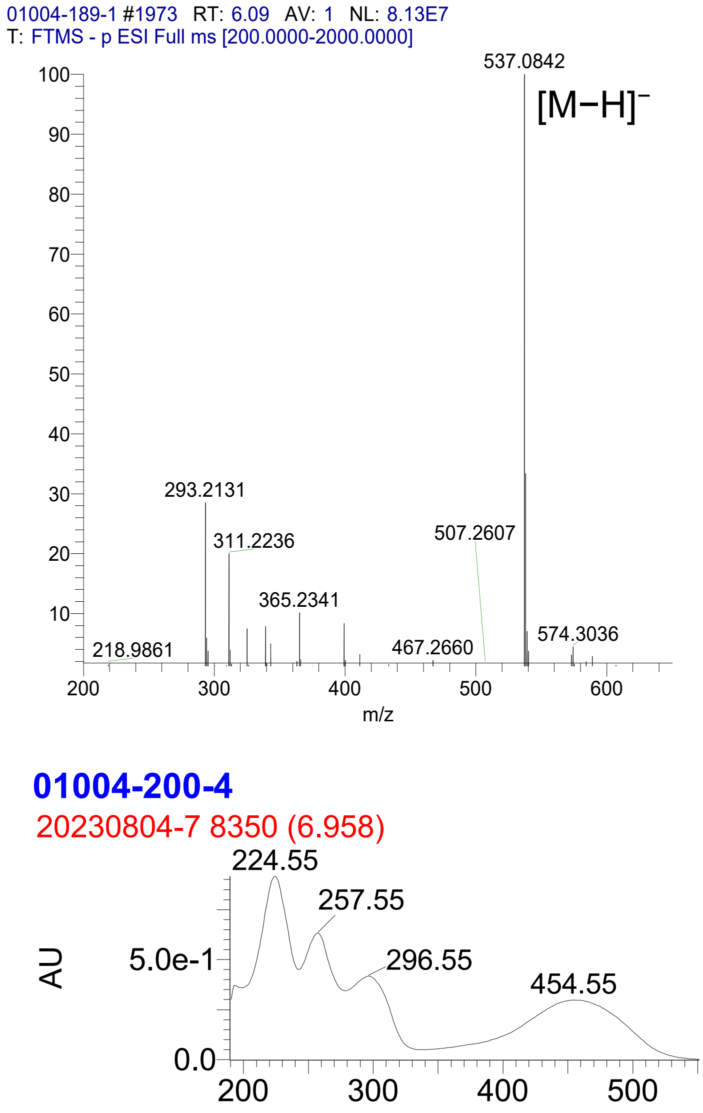

Figure S12. HRESIMS negative mode (top) and UV (bottom) spectra of (+)-skyrin (**4**).

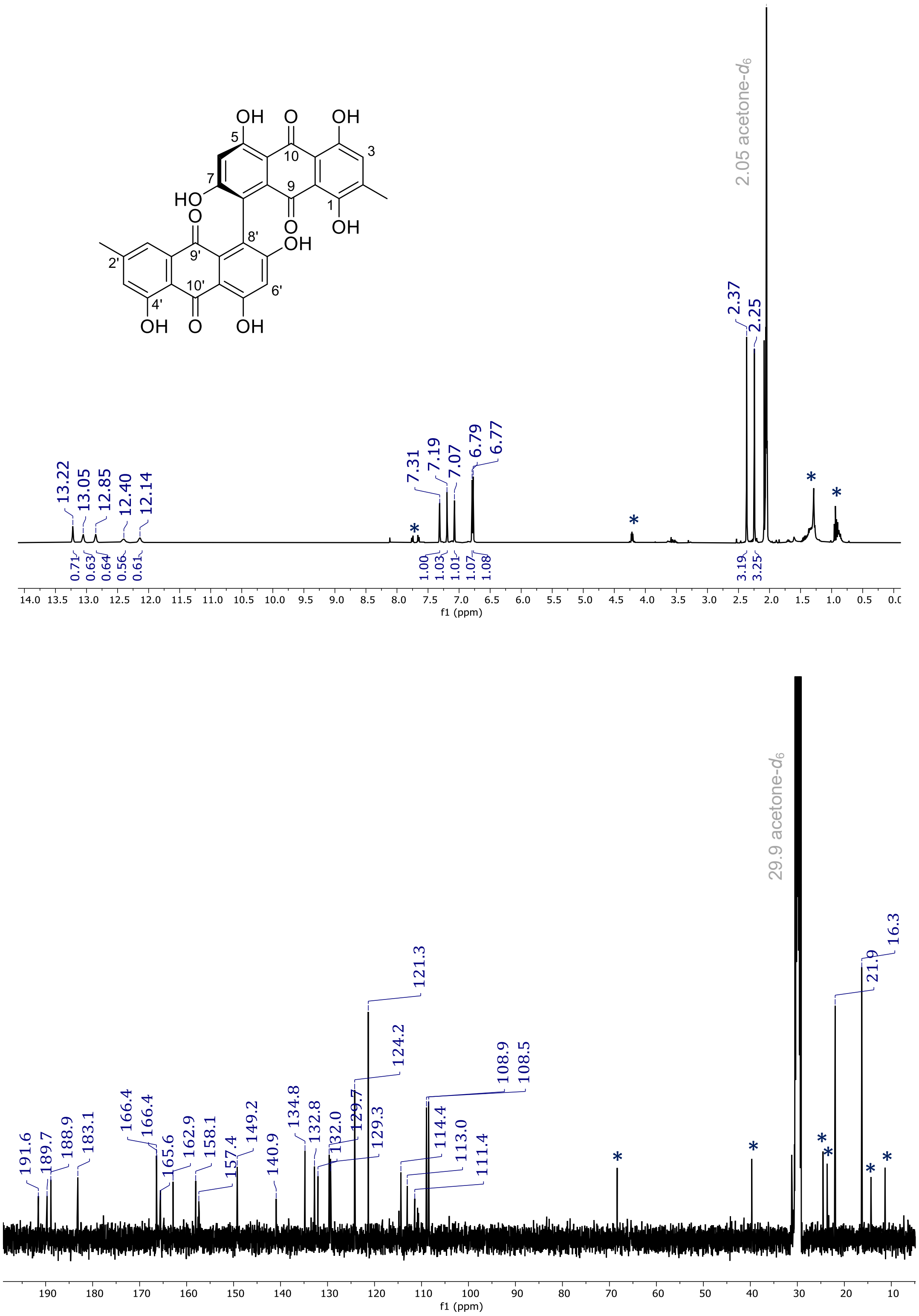

Figure S13. ^{1}H (top) and ^{13}C (bottom) NMR spectra of (+)-aurantioskyrin (**5**) in acetone-d_6 (400 and 100 MHz, respectively). *Impurities.

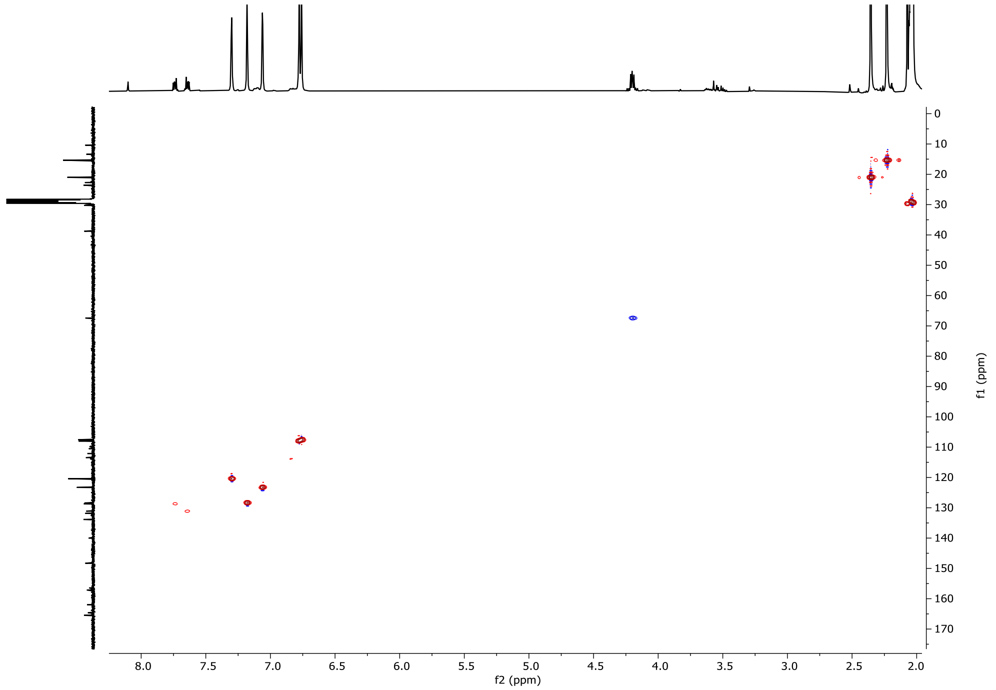

Figure S14. HSCQ spectrum of (+)-aurantioskyrin (**5**) in acetone-d_6 (400 and 100 MHz).

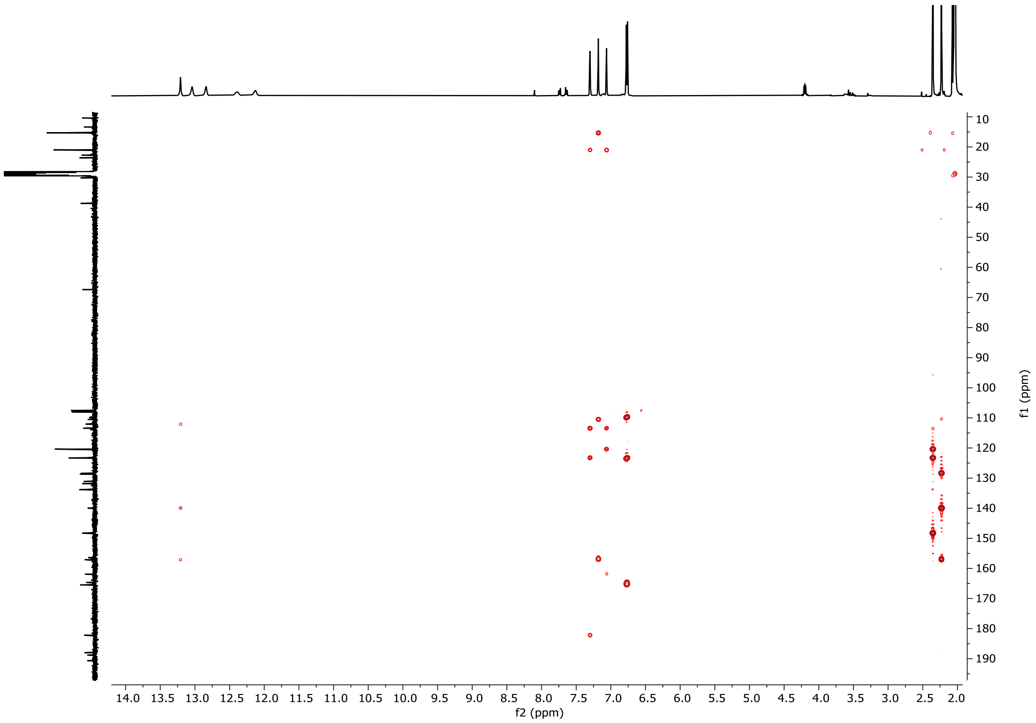

Figure S15. HMBC spectrum of (+)-aurantioskyrin (**5**) in acetone-d_6 (400 MHz).

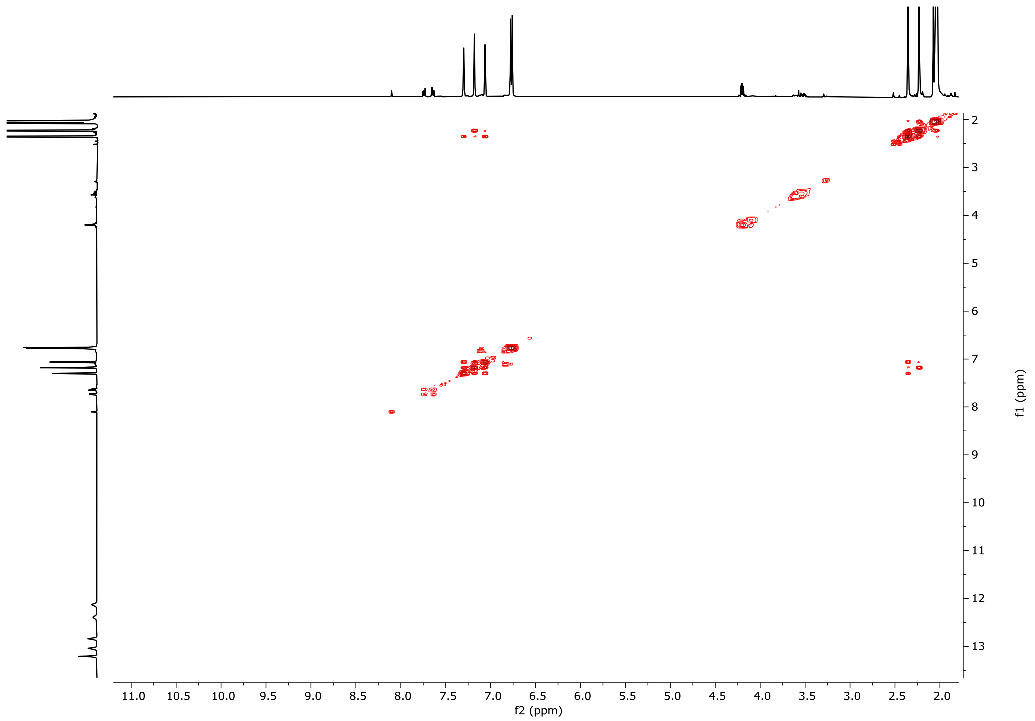

Figure S16. COSY spectrum of (+)-aurantioskyrin (**5**) in acetone-d_6 (400 MHz).

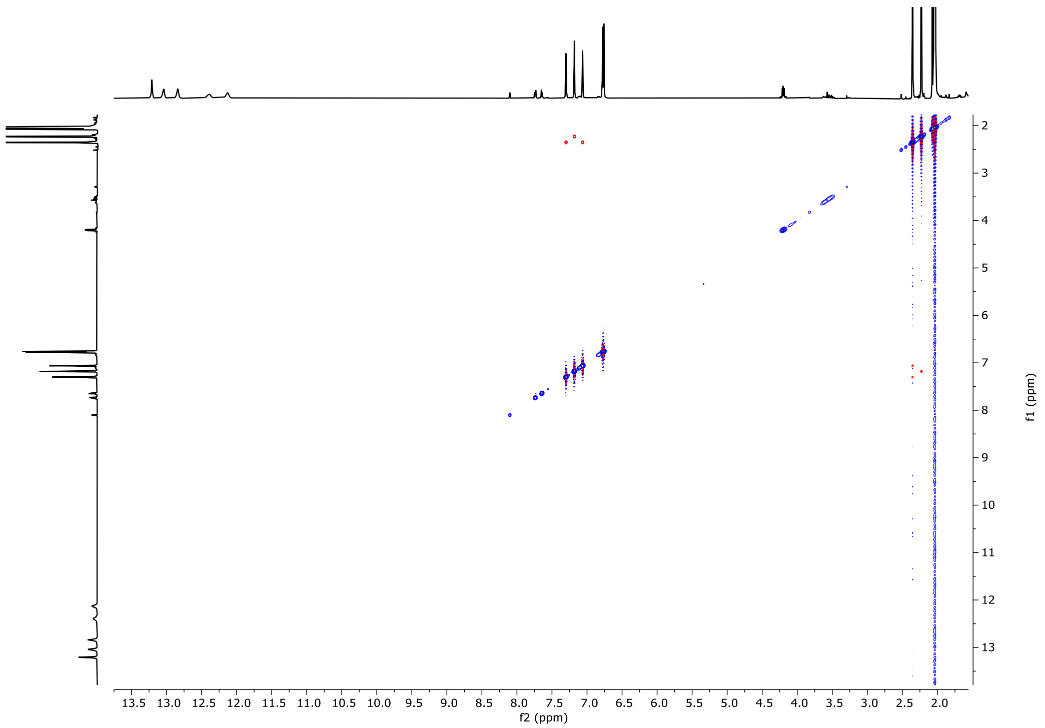

Figure S17. NOESY spectrum of (+)-aurantioskyrin (**5**) in acetone-d_6 (400 MHz).

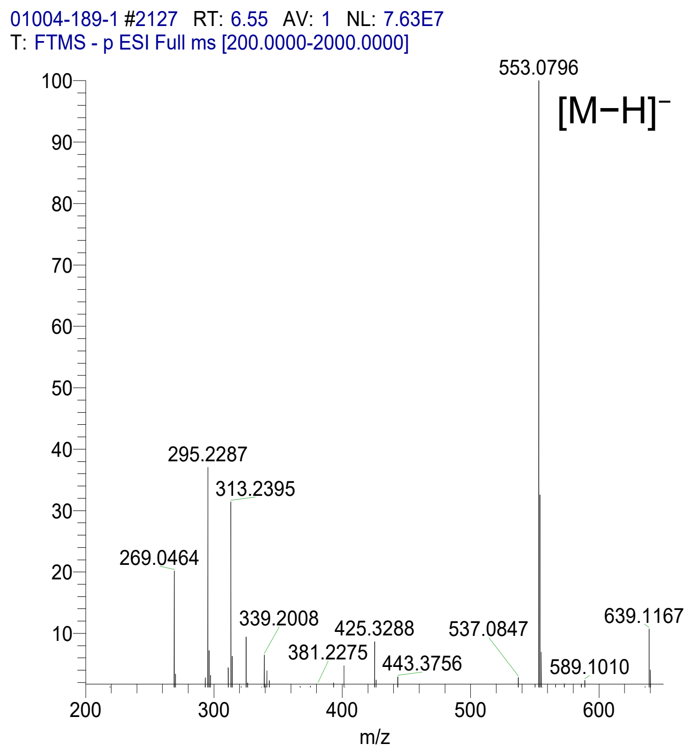

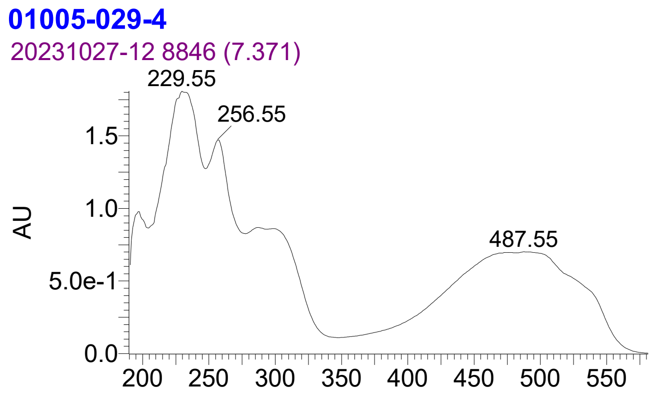

Figure S18. HRESIMS negative mode (top) and UV (bottom) spectra of (+)-aurantioskyrin (**5**).

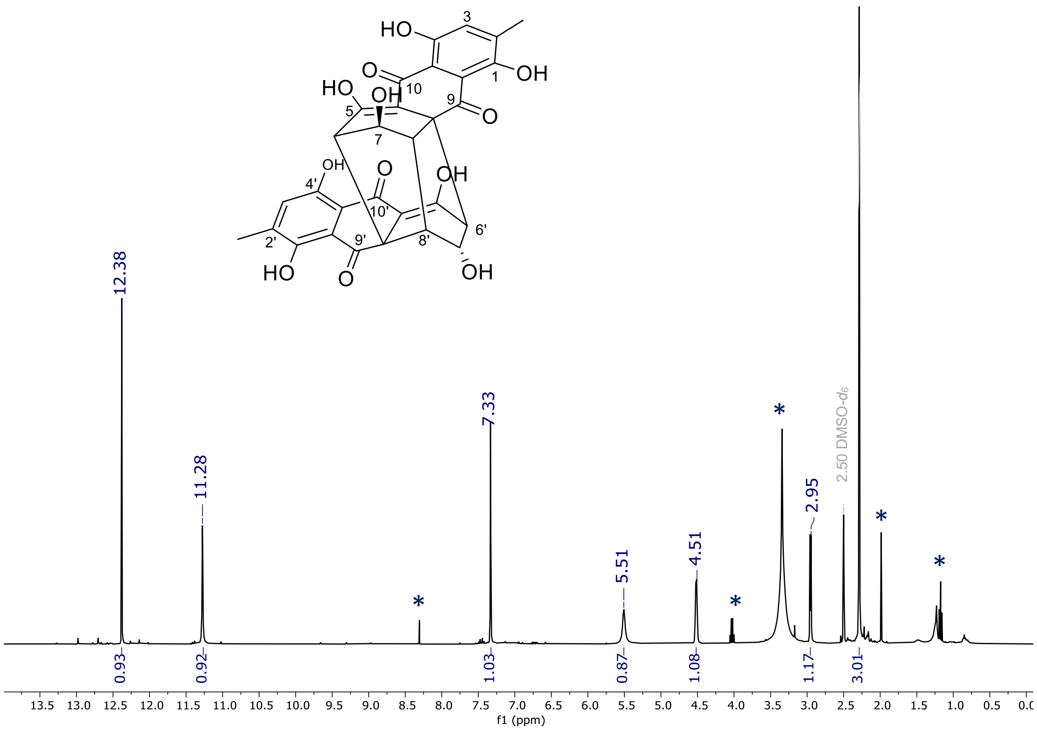

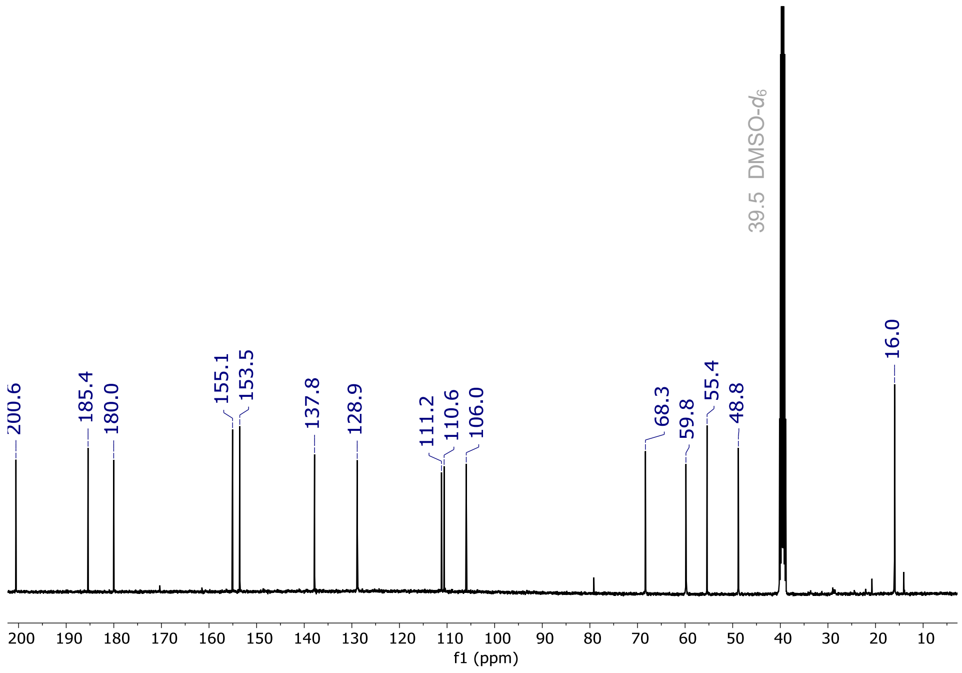

Figure S19. ^{1}H (top) and ^{13}C (bottom) NMR spectra of (−)-luteoskyrin (**6**) in DMSO-d_6 (600 and 150 MHz, respectively). *Impurities.

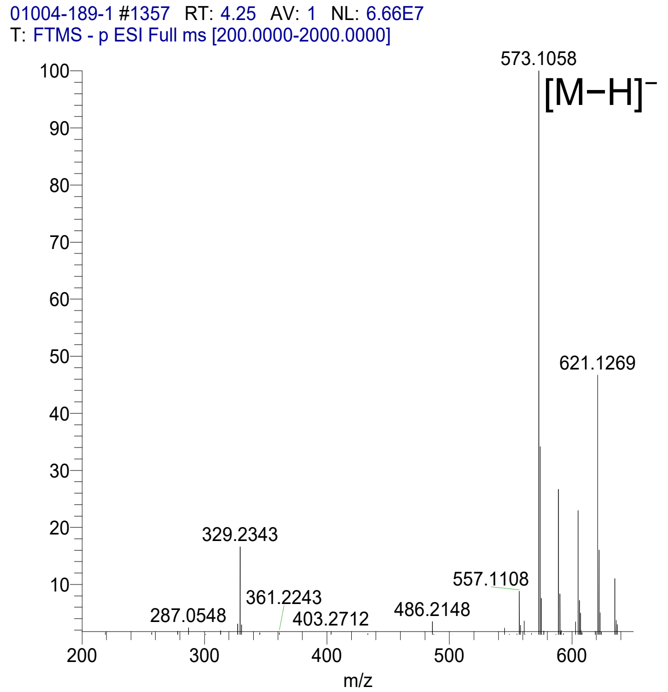

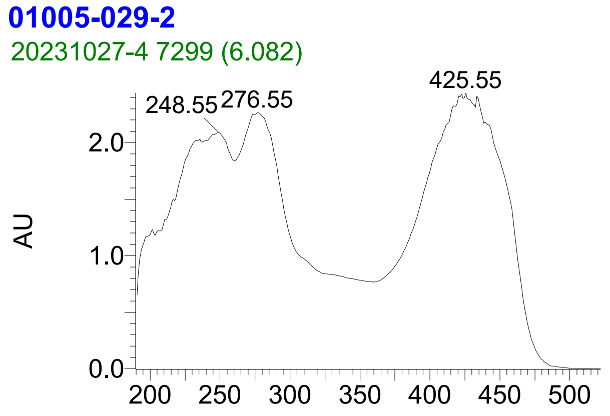

Figure S20. HRESIMS negative mode (top) and UV (bottom) spectra of (−)-luteoskyrin (**6**).

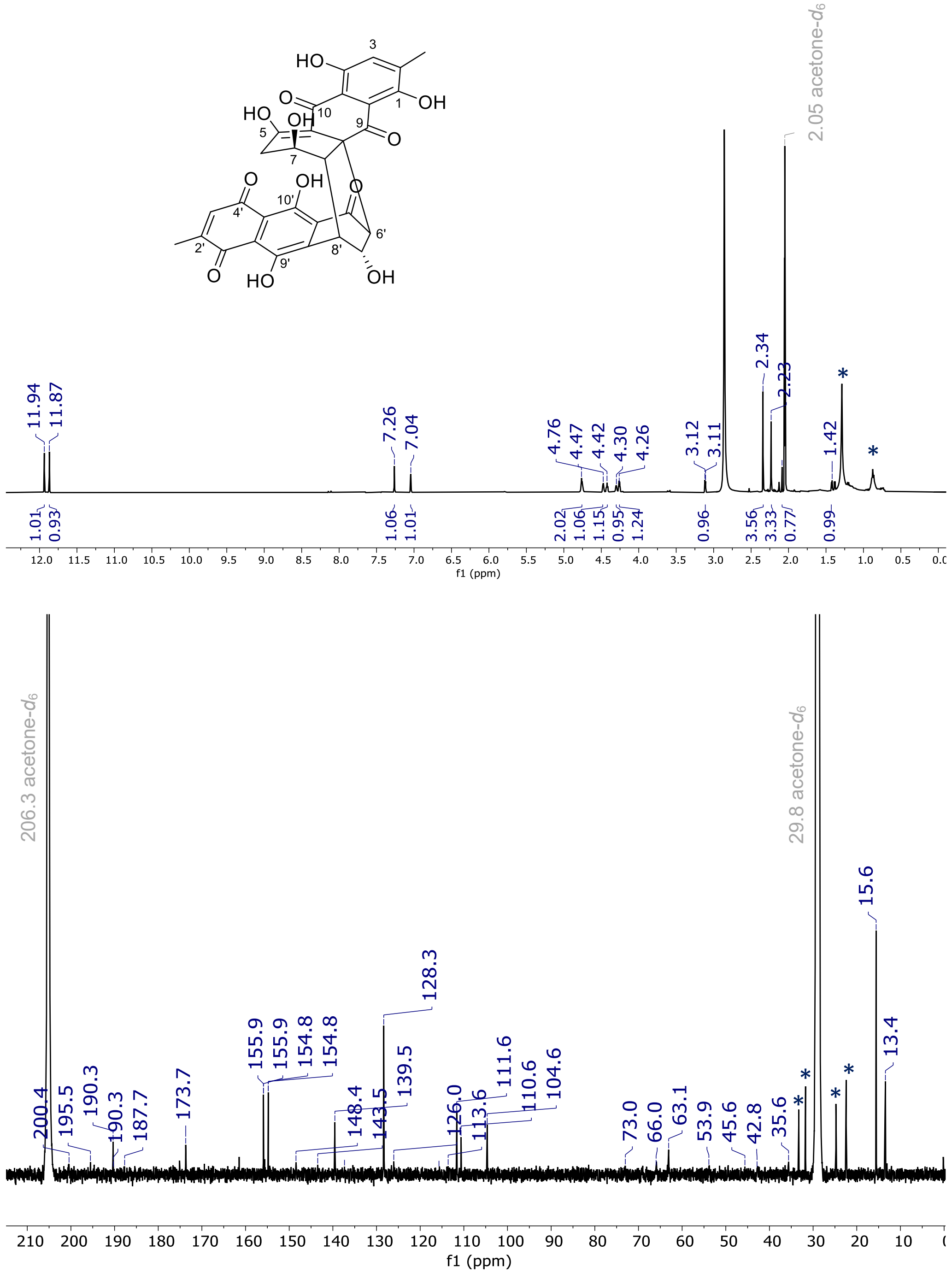

Figure S21. ^{1}H (top) and ^{13}C (bottom) NMR spectra of (−)-rubroskyrin (**7**) in acetone-d_6 (600 and 150 MHz, respectively). *Impurities.

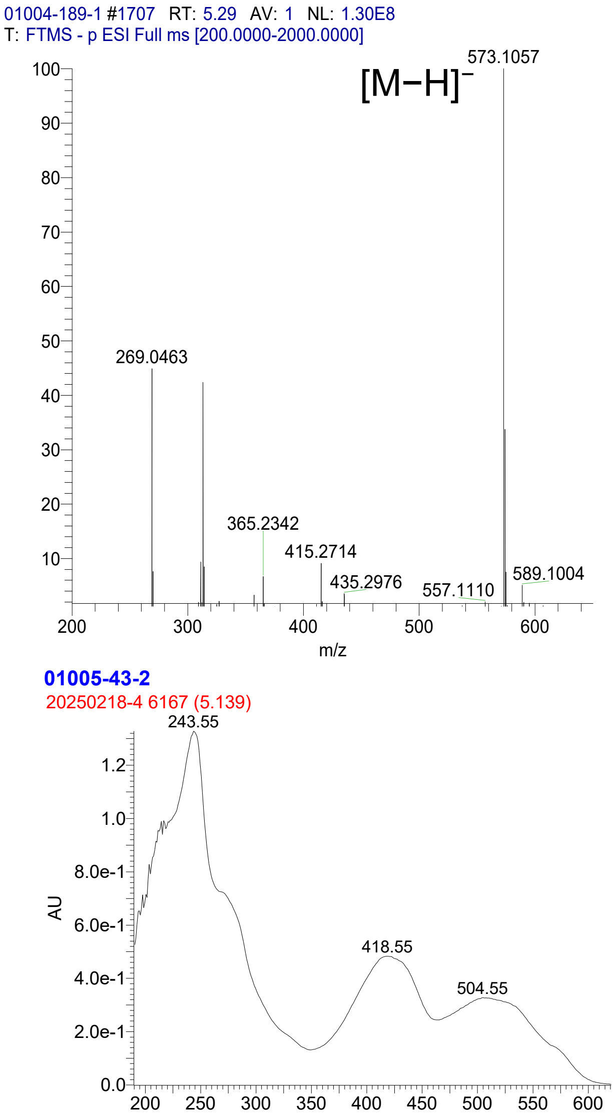

Figure S22. HRESIMS negative mode (top) and UV (bottom) spectra of (−)-rubroskyrin (**7**).

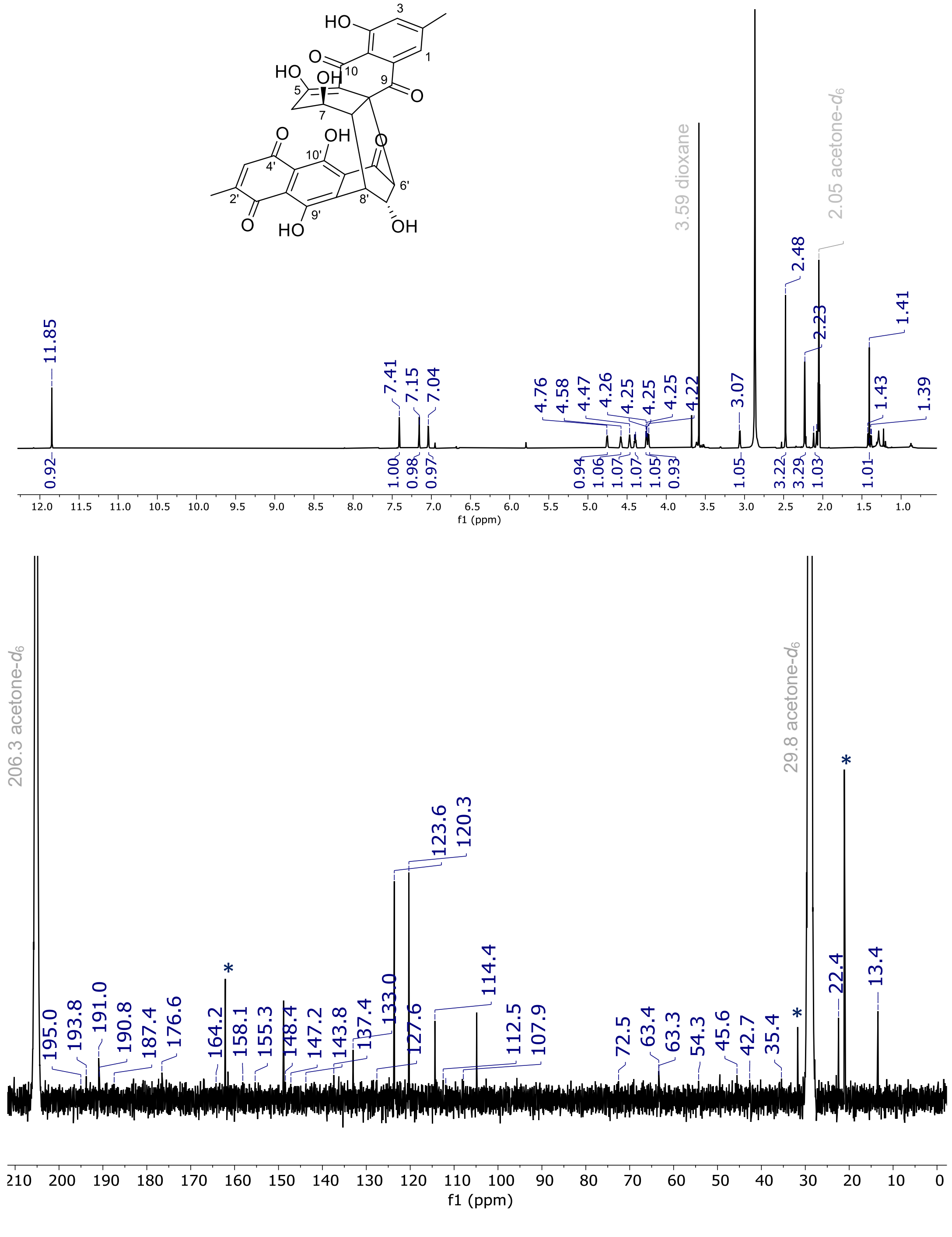

Figure S23. ^{1}H (top) and ^{13}C (bottom) NMR spectra of (−)-deoxyrubroskyrin (**8**) in acetone-d_6 (600 and 150 MHz, respectively). *Impurities.

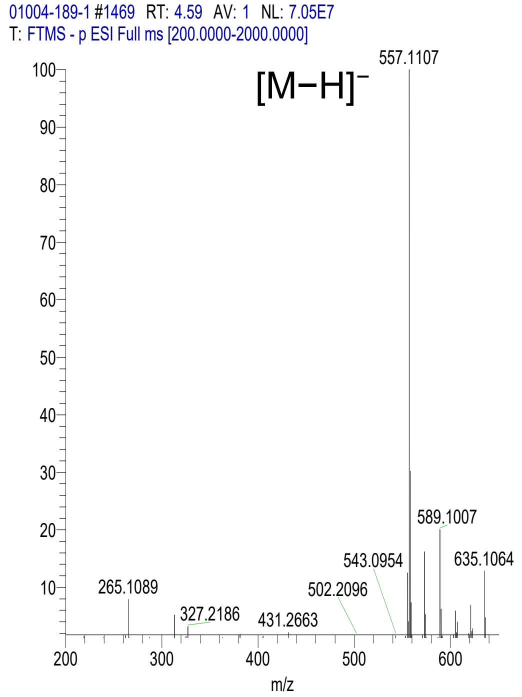

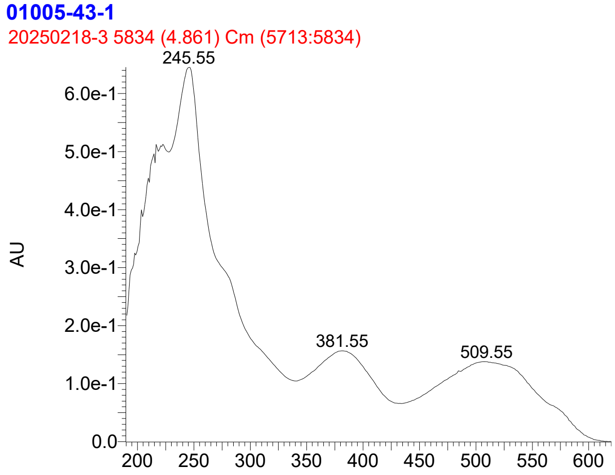

Figure S24. HRESIMS negative mode (top) and UV (bottom) spectra of (−)-deoxyrubroskyrin (**8**).

Table S4. Spectroscopic and spectrometric data of isolated compounds **1−8**.

Islandicin (**1**): red solid; ^{1}H NMR ($CDCl_3$, 400 MHz; Residual solvent peak $CDCl_3$ at δ_H 7.26 ppm). δ_H (ppm) 13.49 (1H, s, 4-OH), 12.34 (1H, s, 5-OH), 12.29 (1H, s, 1-OH), 7.89 (1H, dd, *J*= 1.1, 7.6 Hz, H-6), 7.70 (1H, t, *J*= 8.0 Hz, H-7), 7.31 (1H, dd, *J*= 1.1, 8.4 Hz, H-8), 7.17 (1H, s, H-3) 2.39 (3H, s, 2-CH_3); ^{13}C NMR ($CDCl_3$, 100 MHz; Residual solvent peak $CDCl_3$ at δ_C 77.2 ppm). δ_C (ppm) 190.6 (C-10), 186.7 (C-9), 162.7 (C-4), 158.0 (C-1), 157.9 (C-5), 142.0 (C-2), 136.8 (C-7), 133.7 (C-8a), 129.2 (C-3), 124.7 (C-6), 119.5 (C-8), 116.4 (C-10a), 111.8 (C-9a), 110.8 (C-4a), 16.8 (2-CH_3); HRESIMS *m/z* 269.0463 [M−H]$^-$ (calcd for $C_{15}H_9O_5$, 269.0455, Δ = +2.8 ppm, HDI = 11.0).

Catenarin (**2**): red solid; ^{1}H NMR (DMSO-d_6, 600 MHz; Residual solvent peak at δ_H 2.50 ppm). δ_H (ppm) 13.18 (1H, brs, 8-OH), 12.17 (1H, brs, 1-OH), 7.25 (1H, s, H-3), 7.13 (1H, d, *J*= 2.3, H-5), 6.55 (1H, d, *J* = 2.2 Hz, H-7), 2.25 (3H, s, 2-CH_3); ^{13}C NMR (DMSO-d_6, 150 MHz; Residual solvent peak DMSO-d_6 at δ_C 39.5 ppm). δ_C (ppm) 187.6 (C-9), 186.3 (C-10), 166.1 (C-8), 164.6 (C-6), 156.7 (C-1), 156.1 (C-4), 139.7 (C-3), 134.8 (C-5a), 129.2 (C-3), 111.6 (C-9a), 110.2 (C-4a), 108.9 (C-8a), 108.7 (C-7), 108.3 (C-5), 15.9 (2-CH_3); HRESIMS *m/z* 285.0414 [M−H]$^-$ (calcd for $C_{15}H_9O_6$, 285.0405, Δ = +3.3 ppm, HDI = 11.0)

(+)-Iridoskyrin (**3**): red solid; $[\alpha]_D^{20}$ +3230 (*c* 0.02, 1,4-dioxane); ECD (*c* 2.3 M, 1,4-dioxane) λ_{max} (mdeg): 200 (+0.19), 258 (+2.25), 290 (−3.71), 319 (+5.69), 358(−13.49), 386 (−4.38), 441 (−40.37), 552 (+552), 600 (+0.38) nm; ^{1}H NMR ($CDCl_3$, 400 MHz; Residual solvent peak $CDCl_3$ at δ_H 7.26 ppm). δ_H (ppm) 13.09 (2H, s, 5-OH/5'-OH), 12.90 (2H, s, 4-OH/4-OH'), 12.38 (2H, s, 1-OH/ 1'-OH), 7.35-731 (4H, m, H-7/H-7'/H-6/H-6'), 7.12 (2H, s, H-3/H-3'), 2.28 (6H, s, 2-CH_3/2'-CH_3); ^{13}C NMR ($CDCl_3$, 100 MHz; Residual solvent peak $CDCl_3$ at δ_C 77.2 ppm). δ_C (ppm) 190.8 (C-10/C-10'), 187.2 (C-9/C-9'), 162.7 (C-5/C-5'), 157.8 (C-1/C-1'), 157.5 (C-4/C-4'), 142.0 (C-2/C-2'),138.7 (C-7/C-7'), 137.0 (C-8a/C-8a'), 129.6 (C-8/C-8'), 128.7 (C-3/C-3'), 124.5 (C-6/C-6'), 116.7 (C-1a/C-1a'), 112.0 (C-5a/C-5a'), 110.8 (C-10a/C-10a'), 16.8 (2-CH_3/2'-CH_3). HRESIMS *m/z* 537.0844 [M−H]$^-$ (calcd for $C_{30}H_{17}O_{10}$, 537.0827, Δ = +3.1 ppm, HDI = 22.0).

(+)-Skyrin (**4**): red solid; $[\alpha]_D^{20}$ +402 (*c* 0.13, 1,4-dioxane); ECD (*c* 2.5 M, 1,4-dioxane) λ_{max} (mdeg): 200 (+0.37), 327 (−5.41), 345 (−4.88), 394(−10.87), 507 (+13.37), 560 (+0.51), 600 (−0.05) nm;^{1}H NMR (DMSO-d_6, 600 MHz; Residual solvent peak DMSO-d_6 at δ_H 2.50 ppm). δ_H (ppm)12.80 (2H, s, 5-OH/5-OH'), 12.09 (2H, s, 7-OH/7'-OH), 7.25 (2H, s, H-1/H-1'), 7.10 (2H, s, H-3/H-3'), 6.63 (2H, s, H-6/H-6'), 2.30 (6H, s, 2-CH_3/2'-CH_3); ^{13}C NMR (DMSO-d_6, 150 MHz; Residual solvent peak DMSO-d_6 at δ_C 39.5 ppm). δ_C (ppm) 189.1 (C-10/C-10'), 182.2 (C-9/C-9'), 164.5 (C-5/C-5'/C-7/C-7'), 161.0 (C-4/C-4'), 148.0 (C-2/C-2'), 133.3 (C-9a/C-9a'), 131.2 (C-8a/C-8a'), 123.7 (C-8/C-8'), 123.5 (C-3/C-3'), 120.4 C-1/C-1'), 113.3 (C-4a/4a'), 108.5 (C-5a/C-5a'), 107.1 (C-6/C-6'), 21.5 (2-CH_3/2'-CH_3). HRESIMS *m/z* 537.0842 [M−H]$^-$ (calcd for $C_{30}H_{17}O_{10}$, 537.0827, Δ = +2.8 ppm, HDI = 22.0).

(+)-Aurantioskyrin (**5**): red solid; $[\alpha]_D^{20}$ +1655 (*c* 0.02, 1,4-dioxane); ECD (*c* 2.3 M, 1,4-dioxane) λ_{max} (mdeg): 200 (+0.37), 260 (−0.03), 426 (−30.27), 535 (+19.05), 600 (+0.08) nm; ^{1}H NMR (acetone-d_6, 400 MHz; Residual solvent peak acetone-d_6 at δ_H 2.05 ppm). δ_H (ppm) 13.22 (1H, s, 4-OH), 13.05 (1H, s, 5'-OH), 12.85 (1H, s, 5-OH), 12.40 (1H, s, 4'-OH), 12.14 (1H, s, 1-OH), 7.31 (1H, s, H-1'), 7.19 (1H, s, H-3'), 7.07 (1H, s, H-3), 6.78 (1H, s, H-6'), 6.77 (1H, s, H-6), 2.37 (3H, s, 2'-CH_3), 2.24 (3H, s, 2-CH_3); ^{13}C NMR (acetone-d_6, 100 MHz; Residual solvent peak acetone-d_6 at δ_C 29.9 and 206.6 ppm). δ_C (ppm) 191.6 (C-10'), 189.7 (C-10), 188.9 (C-9'), 183.1 (C-9), 166.4 (C-7/C-7'), 165.6 (C-5/C5'), 162.9 (C-4'), 158.1 (C-1), 157.4 (C-4), 149.2 (C2'), 140.9 (C-2), 134.8 (C-8a) 132.8 (C-8a'), 132.0 (C-9a'), 129.7 (C-3), 129.3 (C-8/C-8'), 124.2 (C-3'), 121.3 (C-1'), 114.4 (C-9a), 113.0 (C-4a'),111.4 (C-4a), 108.9 (C-10a/10a'), 108.5 (C-6'/C-6), 21.9 (2'-CH_3), 16.3 (2-CH_3). HRESIMS *m/z* 553.0796 [M−H]$^-$ (calcd for $C_{30}H_{17}O_{11}$, 553.0776, Δ = +3.2 ppm HDI = 22.0).

(−)-Luteoskyrin (**6**): orange solid; $[\alpha]_D^{20}$ −335 (*c* 0.04, 1,4-dioxane); ECD (*c* 2.3 M, 1,4-dioxane) λ_{max} (mdeg): 200 (−0.33), 250 (−3.15), 260 (−9.17), 302 (+37.66), 329 (−15.41), 341 (+21.44), 372 (−3.34), 446 (−3.81), 539 (+0.37), 600 (−0.27) nm; ^{1}H NMR (DMSO-d_6, 600 MHz; Residual solvent peak DMSO-d_6 at δ_H 2.50 ppm). δ_H (ppm) 12.38 (2H, s, 4-OH/4'-OH),11.28 (2H, s, 1-OH/1'-OH), 7.33 (2H, s, H-3/H-3'), 5.51 (2H, bs, OH-7/OH-7'), 4.54 (2H, dd, *J*= 2.6, 5.9 Hz, H-7/H-7'), 3.34 (2H, brs, H-6/H-6'), 2.95 (2H, dd, *J*= 1.4, 6.5 Hz, H-8/H-8'), 2.29 (6H, s, 2-CH_3/2'-CH3); ^{13}C NMR (DMSO-d_6, 150 MHz; Residual solvent peak DMSO-d_6 at δ_C 39.5 ppm). δ_C (ppm) 200.5 (C-10/C-10'), 185.4 (C-5/C-5'), 180.0 (C-9/C-9'), 155.1 (C-1/C-1'), 153.5 (C-4/C-4'), 137.8 (C-3/C-3'), 128.9 (C-2/C-2'), 111.2 (C-9a/C-9a'), 110.6 (C-4a/C-4a'), 106.0 (C5a-C5a'), 68.3 (C-7/C-7'), 59.8 (C-6/C-6'), 55.4 (C-8a/C-8a'), 48.8 (C-8/C-8'), 16.0 (2-CH_3/2'-CH_3). HRESIMS *m/z* 573.1058 [M−H]$^-$ (calcd for $C_{30}H_{21}O_{12}$, 573.1038, Δ = +3.4 ppm, HDI = 20.0).

(−)-Rubroskyrin (7): red crystalline solid; $[\alpha]_D^{20}$ −480 (*c* 0.005, 1,4-dioxane); ECD (*c* 3.5 M, 1,4-dioxane) λ_{max} (mdeg): 200 (+0.76), 220 (+6.15), 260 (−3.04), 324 (+48.18), 387 (−1.83), 421 (−2.61), 477 (−13.88), 520 (−8.27), 600 (−2.08) nm; ^{1}H NMR (acetone-d_6, 600 MHz; Residual solvent peak acetone-d_6 at δ_H 2.05 ppm). δ_H (ppm) 11.94 (1H, s, 4-OH, 11.87 (1H, s, 1-OH), 7.26 (1H, s, H-3'), 7.04 (1H, s, H-3), 4.76 (1H, s, 7-OH/7'-OH), 4.47 (1H, m, H-7'), 4.42(1H, m, H-7), 4.3-4.26 (1H, m, H-8'/H-8), 3.12 (1H, dd, *J*= 4.7, 1.7 Hz, H-6'), 2.34 (3H, d, *J*= 0.92 Hz, 2'-CH_3), 2.23 (3H, J= 1.53 Hz, 2-CH_3), 2.09 (1H, m, H-6a), 1.40 (1H, d, *J*= 4.13 Hz, H-6b); ^{13}C NMR (acetone-d_6, 175 MHz; Residual solvent peak acetone-d_6 at δ_C 29.9 and 206.6 ppm). δ_C (ppm) 200.4 (C-9), 195.5 (C-5'), 190.3 (C-4'), 190.3 (C-1'), 187.7 (C-5), 173.7 (C-10), 155.9 (C-4), 155.9 (C-10'), 154.8 (C-1), 154.8 (C-9'), 148.4 (C-2'), 143.5 (C-2), 139.5 (C-8a'), 137.3 (C-3'), 128.8 (C-3), 126.0 (C-5a),115.7 (C-9a'), 113.6 (C-9a), 111.6 (C-4a), 110.6 (C-4a'), 104.6 (C-5a), 73.0 (C-7), 66.0 (C-7'), 63.1 (C-6'), 53.9 (C-8), 45.6 (C-6), 42.8 (C-8a), 35.6 (C-8'), 15.6 (2'-CH_3), 13.4 (2-CH_3). HRESIMS *m/z* 573.1057 $[M-H]^-$ (calcd for $C_{30}H_{21}O_{12}$, 573.1038, Δ = +3.2 ppm, HDI = 20.0).

(−)-Deoxyrubroskyrin (8): purple solid; $[\alpha]_D^{20}$ −160 (*c* 0.01, 1,4-dioxane) ECD (*c* 3.6 M, 1,4-dioxane) λ_{max} (mdeg): 200 (+0.45), 222 (−3.08), 257 (−3.04), 314 (+20.45), 364 (−2.06), 408 (−1.71), 479 (−9.50), 550 (−6.20), 600 (−1.05) nm; ^{1}H NMR (acetone-d_6, 600 MHz; Residual solvent peak acetone-d_6 at δ_H 2.05 ppm (quint)). δ_H (ppm) 11.85 (1H, s, 4-OH), 7.41 (1H, d, *J*= 1.6 Hz, H-3'), 7.15 (1H, s, H-1), 7.04 (1H, bs, H-3), 4.76 (1H, s, OH-7'), 4.58 (1H, s, OH-7), 4.47 (1H, m, H-7'), 4.39 (1H, m, H-7), 4.25 (1H, m, H-8'), 4.22 (1H, m, H-8), 3.06 (1H, d, *J*= 4.8 Hz, H-6'), 2.48 (3H, s, 2'-CH_3), 2.23 (3H, s, 2-CH_3), 2.10 (1H, m, H-6a), 1.40 (1H, m, H-6b);^{13}C NMR (acetone-d_6, 175 MHz; Residual solvent peak acetone-d_6 at δ_C δ_C 29.9 and 206.6 ppm). δ_C (ppm) 195.0 (C-9), 193.8 (C-5'), 191.0 (C-4'), 190.8 (C-1'), 187.4 (C-5), 177.6 (C-10), 164.2 (C-4), 158.1 (C-10'), 155.3 (C-9'), 148.4 (C-2), 147.2 (C-2'), 143.8 (C-8a'), 137.4 (C-9a), 133.0 (C-3'), 127.6 (C-5a'), 123.6 (C-3), 120.3 (C-1), 114.4 (C-9a'), 112.5 (C-4a), 111.9 (C-4a'), 107.9 (C-5a), 72.5 (C-7), 63.4 (C-7'), 63.1 (C-6'), 54.3 (C-8), 45.6 (C-6), 42.7 (C-8a), 35.4 (C-8'), 22.4 (2-CH_3), 13.4 (2'-CH_3) HRESIMS *m/z* 557.1107 $[M-H]^-$ (calcd for $C_{30}H_{21}O_{11}$, 557.1089, Δ = +3.2 ppm, HDI = 20.0).

Table S5. GNPS metabolomics annotation in *T. islandicus* M31.

Compound	Observed ion (*m/z*)	Adduct	Molecular formula	Exact mass	Mass accuracy (ppm)
Islandicin (**1**)[b]	269.0465	$[M-H]^-$	$C_{15}H_9O_5$	269.0455	3.5
Catenarin (**2**)[b]	285.0414	$[M-H]^-$	$C_{15}H_9O_6$	285.0405	3.3
(+)-Iridoskyrin (**3**)[b]	537.0849	$[M-H]^-$	$C_{30}H_{17}O_{10}$	537.0827	4.1
(+)-Skyrin (**4**)[b]	537.0844	$[M-H]^-$	$C_{30}H_{17}O_{10}$	537.0827	3.1
(+)-Aurantioskyrin (5)[b]	553.0794	$[M-H]^-$	$C_{30}H_{17}O_{11}$	555.0776	3.2
(−)-Luteoskyrin (**6**)[b]	573.1057	$[M-H]^-$	$C_{30}H_{21}O_{12}$	573.1038	3.2
(−)-Rubroskyrin (**7**)[b]	573.1058	$[M-H]^-$	$C_{30}H_{21}O_{12}$	573.1038	3.4
(−)-Deoxyrubroskyrin (**8**)[b]	557.1107	$[M-H]^-$	$C_{30}H_{21}O_{11}$	557.1089	3.2
Rugulosin (**9**)[a]	541.1162	$[M-H]^-$	$C_{30}H_{21}O_{10}$	541.1140	4.0
Ustilaginoidin P (**10**)[a]	545.1107	$[M+H_2O-H]^-$	$C_{29}H_{21}O_{11}$	545.1089	3.2
Auroskyrin (**11**)[a]	521.0898	$[M-H]^-$	$C_{30}H_{17}O_9$	521.0878	3.8

[a]Annotated by SIRIUS; [b]Manually annotated

Table S6. Biological activity reported for compounds **1-8**.

Compound	Reported Biological activity	Reference
Islandicin (**1**)	Antibacterial	Sadorn, K.; et al. 2019
Catenarin (**2**)	Phytotoxic[2]	Stark, A. A.; et al. 1978
(+)-Iridoskyrin (**3**)	Antibacterial[3]	Bouras, N.; et al. 2008
(+)-Skyrin (**4**)	Antibacterial[4]	Martínez-Rodríguez, O. P.; et al. 2025
(+)-Aurantioskyrin (**5**)	(Photo)antibacterial[5]	Manzano, J. A.; et al. 2025
(−)-Luteoskyrin (**6**)	Antifungal[1]	Sadorn, K.; et al. 2019
(−)-Rubroskyrin (**7**)	Antibacterial[1]	Sadorn, K.; et al. 2019
(−)-Deoxyrubroskyrin (**8**)	Not reported	-

REFERENCES

Sadorn, K.; Saepua, S.; Boonyuen, N.; Komwijit, S.; Rachtawee, P.; Pittayakhajonwut, P. Phenolic glucosides and chromane analogs from the insect fungus *Conoideocrella krungchingensis* BCC53666. *Tetrahedron* **2019**, *75*, 3463–3471. DOI: 10.1016/j.tet.2019.05.007.

Stark, A. A.; Townsend, J. M.; Wogan, G. N.; Demain, A. L.; Ghosh, A. C. Mutagenicity and antibacterial activity of mycotoxins produced by *Penicillium islandicum* Sopp and *Penicillium rugulosum*. *J. Environ. Pathol. Toxicol.* **1978**, *2*, 313–324.

Bouras, N.; Strelkov, S. E. The anthraquinone catenarin is phytotoxic and produced in leaves and kernels of wheat infected by *Pyrenophora tritici-repentis*. *Physiol. Mol. Plant Pathol.* **2008**, *72*, 87–95. DOI: 10.1016/j.pmpp.2008.06.001.

Martínez-Rodríguez, O. P.; García-Contreras, R.; Aguayo-Ortiz, R.; Yeverino, I. R.; Fajardo-Hernández, C. A.; Patiño, A. D.; Hernández-Pérez, H. A.; Hernández-Garnica, M.; Figueroa, M. Natural products inhibit bacterial growth and modulate quorum-sensing systems *Las* and *Rhl* in *Pseudomonas aeruginosa* PA14. *Chem. Biodivers.* **2025**, e02040. DOI: 10.1002/cbdv.202502040.

Manzano, J. A. H.; Abanto, J. C. D.; Dayo, B. A. F.; Llames, L. C. J.; Gonzaga, M. G. E.; Brogi, S.; Macabeo, A. P. G. DNA gyrase-inhibitory antimicrobial anthraquinone from the endophytic sordariomycetes fungus *Diaporthe perseae*. *Arch. Microbiol.* **2025**, *207*, 239. DOI: 10.1007/s00203-025-04429-6.

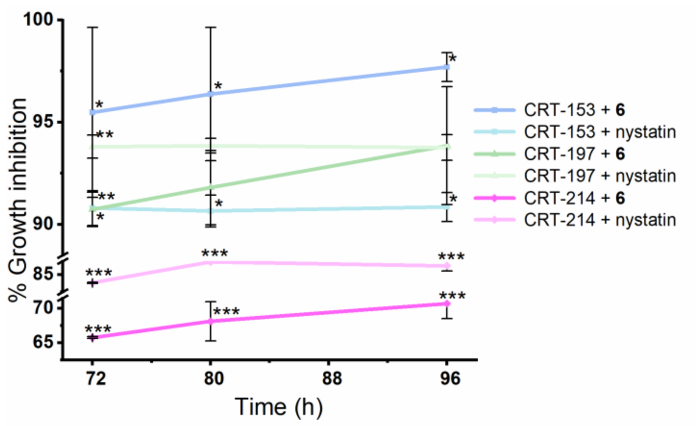

Figure S25. Growth inhibition of *Fusarium* species by (−)-luteoskyrin (**6**) at 10 µg/mL and over 96 hours. Data corresponds to the mean ± SEM of three technical replicates. * $p<0.05$; ** $p<0.05$; *** $p<0.005$

Table S7. MIC and IC_{50} values of (−)-luteoskyrin (**6**) against agave pathogens.[a]

	***F. incarnatum* CRT-153**	***F. incarnatum* CRT-197**	***F. oxysporum* CRT-214**
MIC (μM)	17.4	8.7	34.9
IC_{50} μM	8.7±0.4	4.4±0.6	16.0±2.6
MIC nystatin (μM)[b]	21.6	21.6	53.9

[a]Values were determined using three technical replicates. [b]Positive control.

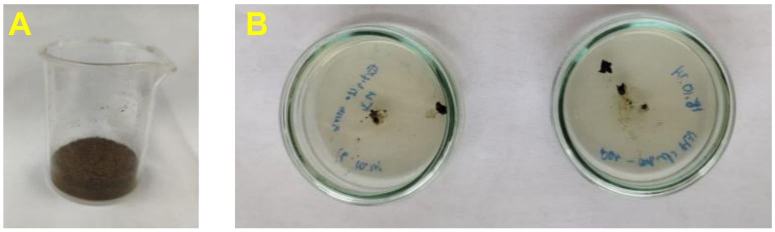

Figure S26. (A) Fungal mycelial formulation of inactivated mycelia of *T. islandicus* M31. (B) Sterility test of the formulation after 7 days of incubation at 30°C and 12/12 h light-dark cycle.

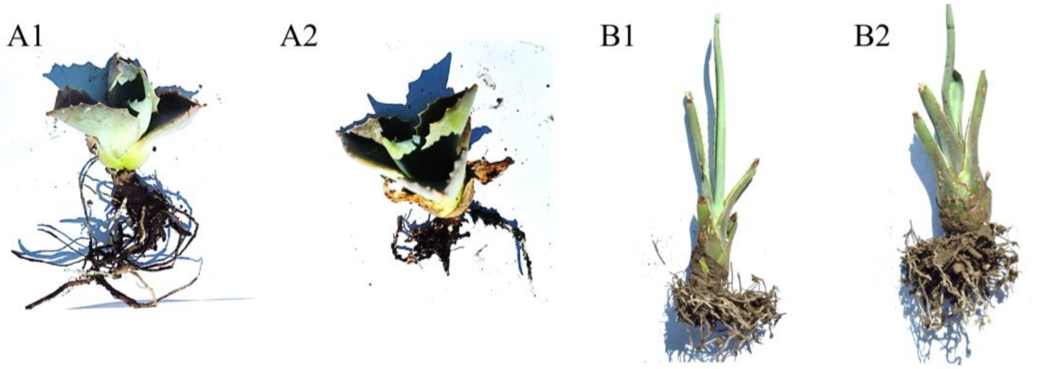

Figure S27. Toxicity test of the fungal mycelial formulation of *T. islandicus* M31 in *A. potatorum* and *A. angustifolia* at (A1 and B1) 1 mg/cm^3 and (A2 and B2) 0.75 mg/cm^3. The experiments were performed with three technical replicates

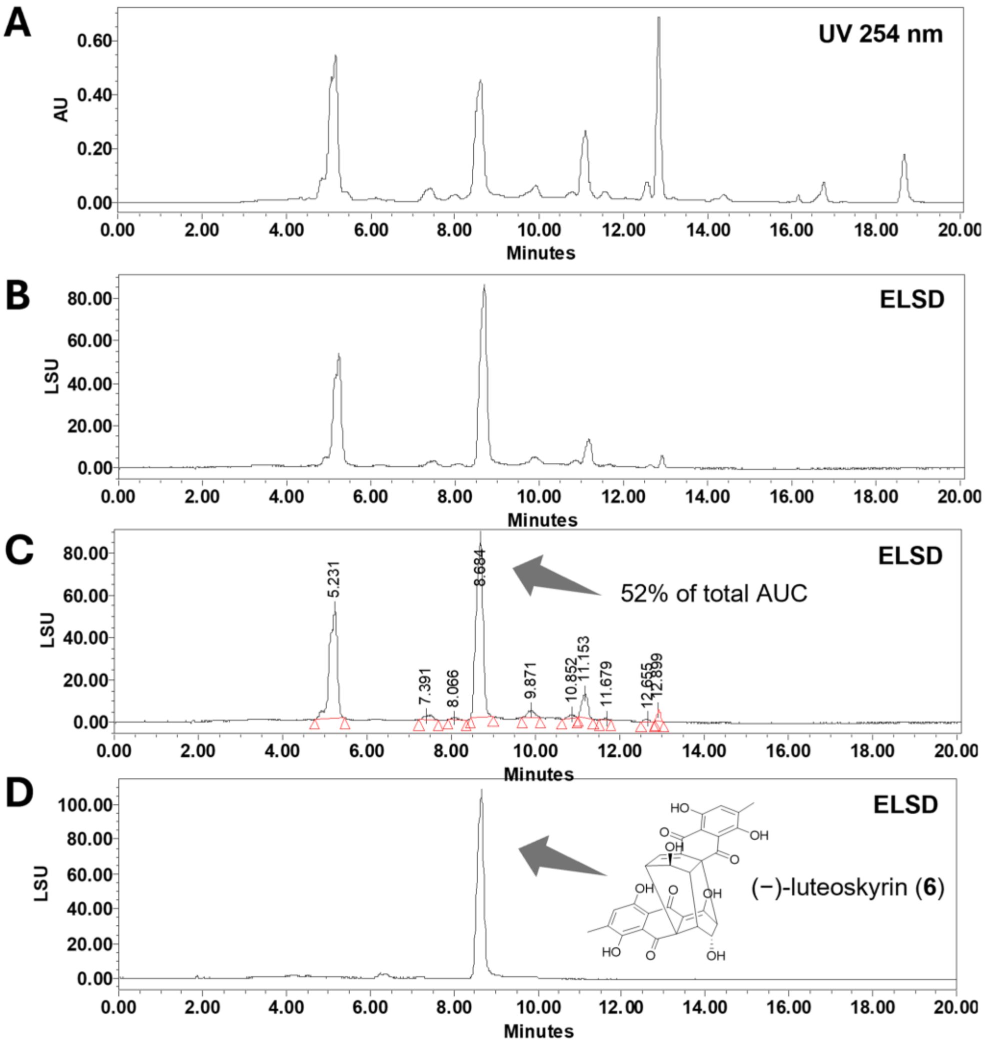

Figure S28. Relative quantification of **6** ion the fungal mycelial formulation of *T. islandicus* M31. HPLC chromatogram of the organic extract from the fungal mycelial formulation of *T. islandicus* M31 (2 mg/mL) by (A) UV at 254 nm, (B) ELSD, and (C) ELSD with AUC integration; and (D) of the pure (−)-luteoskyrin (**B**) by ELSD. Chromatographic conditions: Gemini C18 column (5 µm, 110 Å, 250 × 4.6 mm i.d.), gradient from 40:60 to 100:0 of CH_3CN-0.1% aqueous formic acid in 7 min at 1 mL/min (run time: 20 min), injection volume, 20 µL.
